# LepR‐Lineage Cells Represent the Main Target of the Nociceptive CGRP Pathway in White Adipose Tissue Browning

**DOI:** 10.1002/advs.78714

**Published:** 2026-11-09

**Authors:** Yu‐Ting Yen, May Chien, Yan‐Tung Kiu, Ru‐Sing Lee, Hui‐Teng Cheng, Shuei‐Liong Lin, Dong‐Chuan Wu, I‐Hsien Teng, Yi‐Wen Lin, Yi‐Hung Chen, Mien‐Chie Hung, Shih‐Chieh Hung

**Affiliations:** ^1^ Drug Development Center, Institute of Translational Medicine and New Drug Development, School of Medicine China Medical University Taichung Taiwan; ^2^ Department of Internal Medicine National Taiwan University Hospital Hsin‐Chu Branch Zhubei City Taiwan; ^3^ Graduate Institute of Physiology National Taiwan University College of Medicine Taipei Taiwan; ^4^ Graduate Institute of Biomedical Sciences, Research Center for Cancer Biology China Medical University Taichung Taiwan; ^5^ Translational Medicine Research Center China Medical University Hospital Taichung Taiwan; ^6^ Graduate Institute of Acupuncture Science China Medical University Taichung Taiwan; ^7^ Center For Molecular Medicine China Medical University Hospital Taichung Taiwan; ^8^ College of Life Sciences China Medical University Taichung Taiwan; ^9^ Integrative Stem Cell Center China Medical University Hospital Taichung Taiwan; ^10^ Department of Orthopaedics China Medical University Hospital Taichung Taiwan

**Keywords:** adipose progenitors, CGRP signaling, LepR‐lineage cells, nociceptive innervation, thermogenesis, TRP channels, white adipose tissue browning

## Abstract

Sympathetic innervation regulates white adipose tissue (WAT) browning; however, the roles of nociceptive neurons and their associated target cells remain unknown. Here, we used single‐nucleus RNA sequencing to profile adipose populations and genetic lineage tracing to identify LepR‐lineage stromal/progenitor cells contributing to cold‐induced WAT browning. Ablation of these cells using LepR‐Cre‐inducible DTR transgenic mice resulted in a decrease in the percentage of UCP1+ cells under cold conditions. The WAT browning process is controlled by peripheral nociceptive nerve‐derived calcitonin gene‐related peptide (CGRP), which was associated with reduced WAT browning and loss of LepR‐lineage cells during intermittent fasting (IF), but not during cold exposure. Local antagonist experiments targeting the transient receptor potential cation channels TRPV1 and TRPM8 were associated with opposing changes in CGRP immunoreactivity and WAT browning. These findings support a condition‐dependent role for nociceptive CGRP signaling in WAT remodeling.

## Graphical abstract

Regulation of WAT Browning and UCP1 Expression in LepR‐MSCs of Inguinal WAT by Nociceptive CGRP and Sympathetic Nervous System (SNS) Noradrenaline (NE).

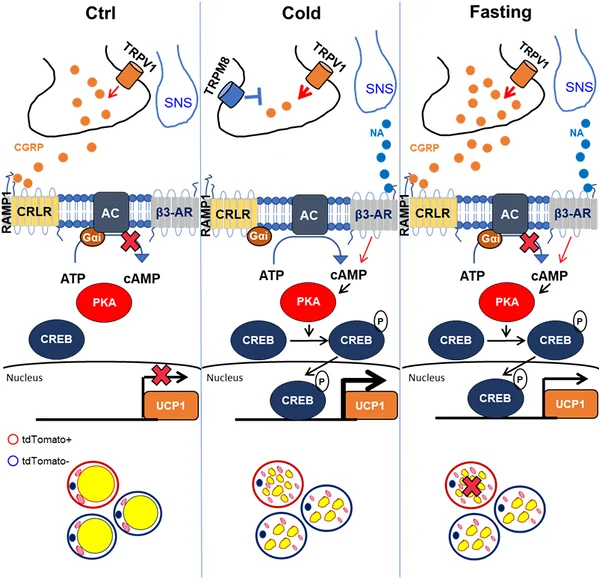

## 1 Introduction

Mammalian adipose tissues are divided into white adipose tissue (WAT) and brown adipose tissue (BAT) based on their function and morphology [1]. WAT acts as an energy store, whereas BAT serves as a heat generator via the mitochondrial uncoupling protein 1 (UCP1) to maintain the core body temperature [2]. A recently identified type of fat, beige or brite, is morphologically and functionally similar to BAT but more developmentally related to WAT [3]. Given its favorable effect on whole‐body energy metabolism, the induction of beige cells from WAT, known as WAT browning, plays important roles in glucose homeostasis, insulin sensitivity, and lipid metabolism [4]. Although many agents capable of causing WAT browning have been identified and extensively tested in human and animal models, the mechanism underlying the browning of WAT is still not fully understood [5]. Therefore, there is an urgent need to discover new methods for inducing WAT browning as potential therapeutic strategies for obesity and type 2 diabetes mellitus.

The thermogenic activity of BAT is primarily driven by the sympathetic nervous system (SNS) and mediated by norepinephrine release, which drives adrenergic receptor signaling to enhance the expression of proteins involved in thermogenesis and stimulate intracellular lipolysis [6]. Long‐term sympathetic outflow toward fat deposits also leads to WAT browning [7]. In addition, there are mechanisms underlying adipose‐to‐brain communication. One example involves the adipokine leptin, which is released from the adipose tissue into the circulation and acts on the hypothalamus to regulate appetite and systemic metabolism [8].

Nociceptive neurons are activated by painful stimuli from the body, which trigger a series of electrical signals that travel along afferent nerve fibers to the spinal cord [9, 10]. When these signals reach the spinal cord, the nerve endings release neuropeptides such as calcitonin gene‐related peptide (CGRP) [11], which then activate receptors on other neurons in the spinothalamic tract [12]. Although studies have shown that adipose tissue in hamsters and rats is connected to sensory nerves‐using methods like labeling tissue with antibodies that detect CGRP (a marker for sensory neurons) or selectively damaging WAT sensory nerves with capsaicin [13]—the exact role of these sensory signals in influencing WAT browning is still not fully understood. Recent advances in scientific research utilizing a viral engineering pipeline with AAV9 mutants have revealed the negative effects of somatosensory innervation on lipogenic and thermogenic transcription programs in adipose tissue under thermoneutral conditions [14]. However, the precise role of nociceptive innervation in regulating WAT browning during cold exposure or intermittent fasting (IF), as well as the specific target cells and signaling molecules involved, remain largely unknown.

Transient receptor potential (TRP) channels are multifunctional signaling molecules expressed in a variety of cell and tissue types [15] and are activated by physical (temperature, voltage, pressure, and tension) and chemical stimuli [15]. The mammalian TRP channel superfamily comprises 28 members (27 in humans). Transient receptor potential cation channel subfamily V member 1 (TRPV1) is highly expressed in primary sensory neurons and serves as a major integrator of pain stimuli and a key initiator of neurogenic inflammation [16]. TRPV1 is activated by capsaicin in mammals, exhibits a distinct thermal activation threshold, and is a noxious heat sensor in some mammals, including humans [17]. In contrast, transient receptor potential cation channel subfamily M member 8 (TRPM8) is blocked by warmth [18] and detects cold and menthol in the sensory neurons [19, 20]. TRPM8^–/–^ mice are unable to distinguish between cold and warm conditions and exhibit significantly attenuated cold allodynia [21]. However, the roles of TRPV1 and TRPM8 in the regulation of peripheral CGRP secretion in nociceptive neurons and WAT browning have not been explored.

White adipocytes can undergo transdifferentiation to adopt a beige adipocyte phenotype. Recent studies examined Cre/inducible Cre mouse strains that mark adipocyte, muscle, and mural lineages, and proposed that only smooth muscle actin (*SMA*)‐marked perivascular mural cells tracked the cold‐induced beige lineage [22]. A cell labeling/fate mapping study indicated that another source of beige adipocytes is smooth muscle‐like progenitor cells expressing myosin heavy chain 11 (*Myh11*) [23]. In addition, a perivascular platelet‐derived growth factor receptor‐α (*Pdgfra*)+ cell was identified as a possible source for β3 agonist‐induced beige adipocytes [24]. Leptin‐receptor (LepR)+ mesenchymal stem cells (LepR‐MSCs) represent the main source of bone formed by adult bone marrow [25] and contribute to embryonic and postnatal endochondral ossification [26]. These cells have recently been proposed to produce hematopoietic stem cell regulators and partition into subsets spanning a differentiation continuum [27]. Notably, LepR‐MSCs are heterogeneous and include periarteriolar LepR+ cells poised to undergo osteogenesis and perisinusoidal LepR+ cells poised to undergo adipogenesis during development [28]. However, whether LepR‐MSCs participate in WAT browning during cold exposure and IF has not been explored.

## 2 Results

### 2.1 Comparison of Cold Exposure and Intermittent Fasting in WAT Browning

To characterize WAT browning induced by cold exposure and intermittent fasting (IF), we first compared the physiological, molecular, and histological responses under these conditions. Compared with controls, mice continuously exposed to 6°C for 7 days (Cold group) or subjected to alternate‐day IF for 6 days (IF group) [29] lost body weight (Figure 1a). Rectal temperature increased in the Cold group, whereas IF mice showed higher temperatures than controls at selected measurement time points on Days 7 and 8 (Figure 1b). RT‐qPCR showed marked induction of *Ucp1* mRNA after cold exposure and a smaller increase after IF, whereas immunoblotting showed increased UCP1 protein in both groups compared with controls (Figure 1c,d). H&E staining and PLIN immunofluorescence showed increased proportions of multilocular adipocytes in both groups, with a greater increase following cold exposure (Figure 1e,f). PRDM16, a key regulator of brown/beige adipocyte differentiation, was markedly induced following cold exposure, whereas its induction was substantially lower under IF (Figure 1g). CD137 expression was lower following cold exposure than under IF, consistent with previous reports that CD137 negatively regulates WAT browning during cold exposure [30]. Collectively, these morphological and molecular findings support WAT browning under both conditions, with a more pronounced response to cold exposure.

**FIGURE 1:**
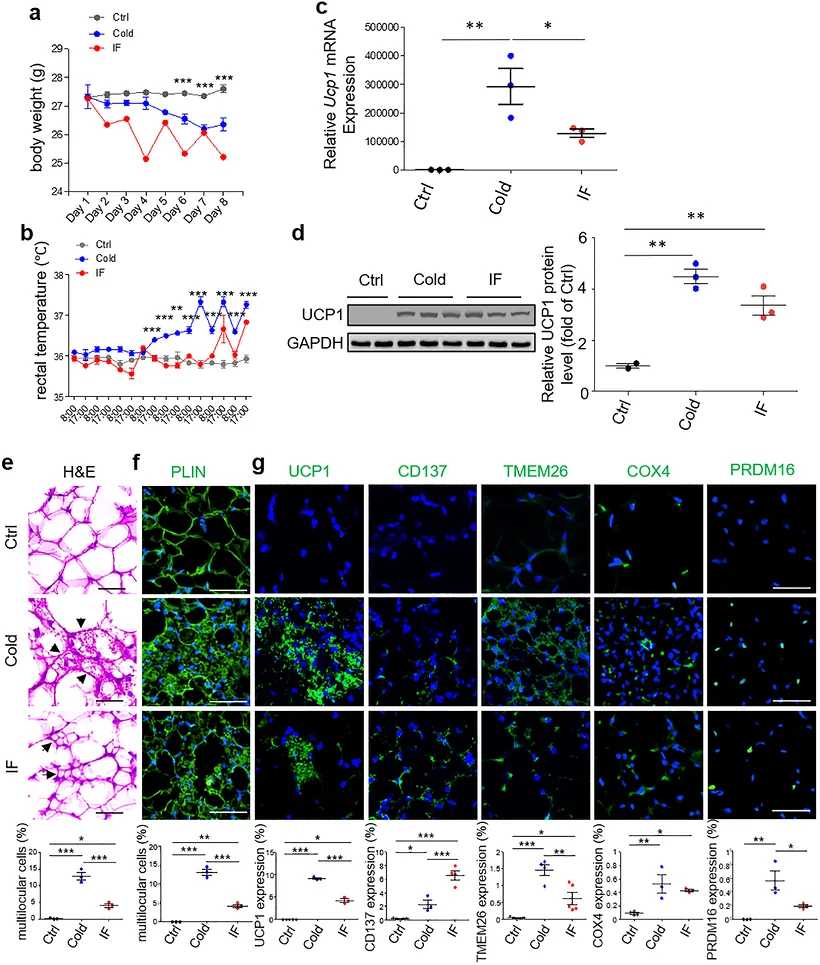
Comparison of cold exposure and intermittent fasting in WAT browning. (a) Body weight and (b) rectal temperature during the 8‐day observation period in Control (Ctrl; 23°C), Cold (6°C), and intermittent fasting (IF; alternating 24‐h fed and fasted periods for three cycles) groups; n = 3 mice per group. Rectal temperature was measured twice daily (08:00 and 17:00). (c) Relative *Ucp1* mRNA expression in iWAT normalized to *Gapdh*. (d) Representative immunoblots and densitometric analysis of UCP1 in iWAT; GAPDH served as the loading control. (e) Representative H&E staining of 5‐µm iWAT sections. Arrowheads indicate multilocular adipocytes. The percentage of multilocular adipocytes was calculated from three high‐power fields per mouse. Enlarged representative views are provided in the Resource Data for Figure 1e. (f) Representative immunofluorescence images of PLIN in iWAT, with quantification of multilocular cells. (g) Representative immunofluorescence images and quantification of UCP1, CD137, TMEM26, COX4, and PRDM16 in iWAT. Each plotted point represents one mouse. Data are mean ± SEM. One‐way ANOVA with Tukey's multiple‐comparison test was used unless otherwise indicated. ^*^ *p* < 0.05, ^**^ *p* < 0.01, ^***^ *p* < 0.001. Scale bars, 50 µm.

### 2.2 snRNA‐Seq Profiles Adipose and Niche Populations During WAT Browning

To characterize adipose cellular heterogeneity and explore transcriptional‐state patterns associated with WAT browning during cold exposure and intermittent fasting (IF), we performed single‐nucleus RNA sequencing (snRNA‐seq). After library‐specific quality control and doublet removal, 3,268 nuclei were retained for downstream analysis (Control, 1,148; Cold, 719; intermittent fasting [IF], 1,401). Unsupervised clustering of the gene‐expression profiles revealed heterogeneity within each cell type, including multiple distinct clusters. Cell annotation combined scCATCH predictions with cluster‐enriched marker genes, canonical marker programs, and published adipose single‐cell atlases [31]. We identified ten cell populations after dataset integration and doublet removal: adipocytes, preadipocytes, white adipocytes, beige adipocytes, stem/progenitor cells, stromal cells, endothelial cells, fibroblasts, B cells, and macrophages (Figure 2a,b). Among the recovered nuclei, macrophage‐annotated nuclei accounted for 16%, 3%, and 7% of the IF, Control, and Cold datasets, respectively, whereas B‐cell‐annotated nuclei represented 3% and 6% of the Control and Cold datasets (Figure 2c). These percentages describe the composition of recovered nuclei. The 3% macrophage fraction in the Control group was compared with published adipose snRNA‐seq data [32]. Segregation of the aggregated UMAP plot showed differences in annotated cell‐state representation, including 41% white adipocytes in Control, 2% beige adipocytes in Cold, and 1% beige adipocytes in IF (Figure 2a–c). Within the recovered nuclei, the Cold dataset exhibited a more apparent beige‐adipocyte‐associated transcriptional signature, providing descriptive evidence of adipogenic‐state heterogeneity across the sampled conditions. To examine adipogenic cell‐state relationships, the stem/progenitor and adipocyte populations in the Control‐plus‐Cold and Control‐plus‐IF datasets were analyzed separately with Monocle3, with scVelo providing complementary RNA‐velocity information. The stem/progenitor region was designated as the pseudotime root, and endogenous *Lepr* expression was overlaid only after trajectory construction to avoid using *Lepr* expression to define the trajectory. Projection of *Lepr* expression highlighted *Lepr*‐expressing nuclei at early positions along the inferred adipogenic trajectory. This analysis illustrated adipogenic transcriptional‐state relationships. These transcriptional‐state patterns (Figure 2d) complement independent LepR‐Cre lineage‐tracing evidence supporting the contribution of LepR‐lineage cells to beige‐adipocyte formation (Figure 3).

**FIGURE 2:**
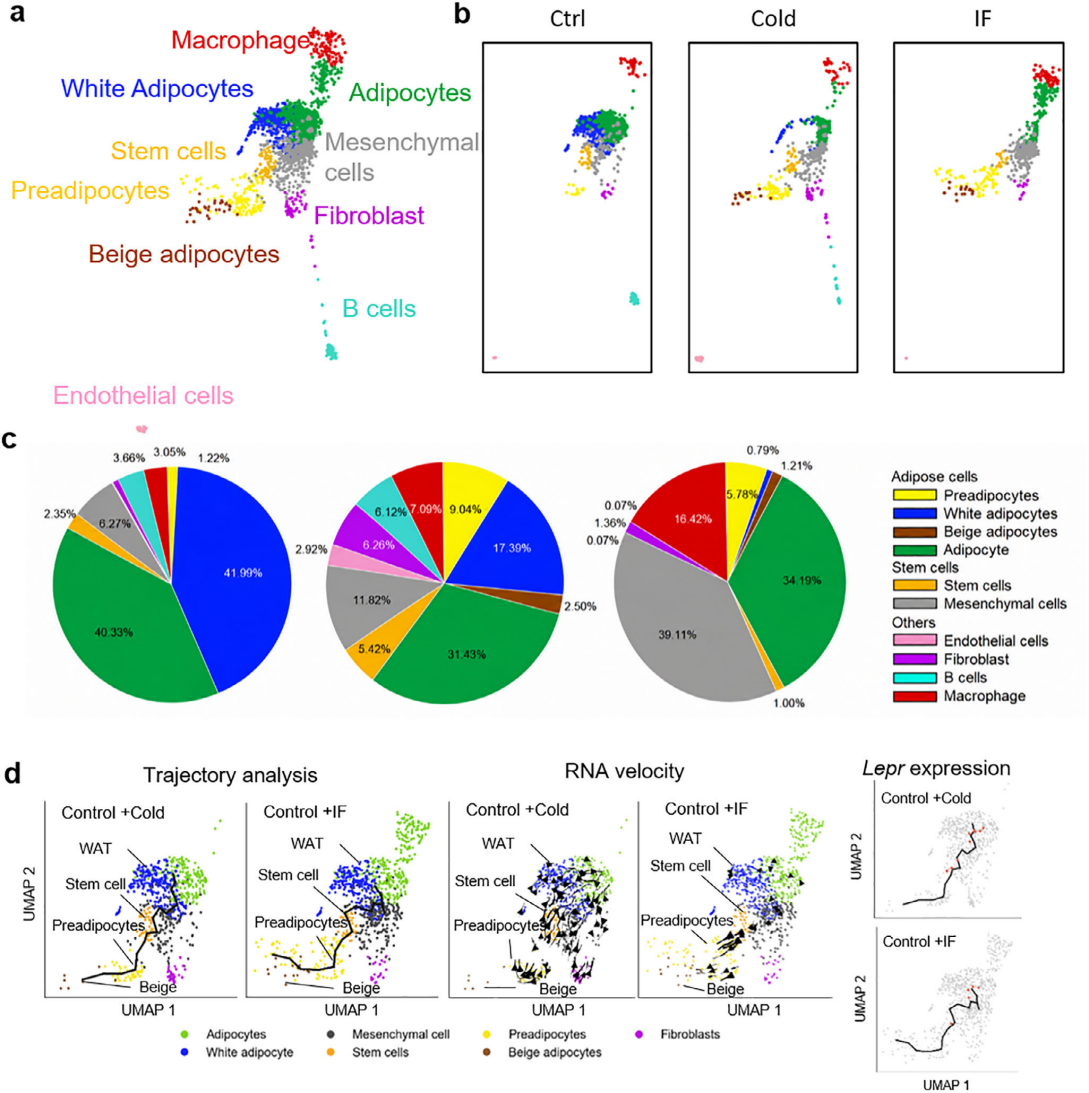
snRNA‐seq profiles iWAT cell states. (a) UMAP of 3,268 nuclei after QC and doublet removal. (b) Distribution of annotated nuclei among the three conditions. (c) Percentage of nuclei in each displayed category. (d) Inferred adipogenic transcriptional‐state trajectories of stem/progenitor and adipocyte populations in Control‐plus‐Cold and Control‐plus‐IF datasets. Monocle3 principal graphs are shown with complementary scVelo RNA‐velocity vectors. A Control stem/progenitor nucleus was designated as the root. Nuclei with detectable endogenous *Lepr* transcripts (raw UMI > 0) were overlaid in red after trajectory inference.

**FIGURE 3:**
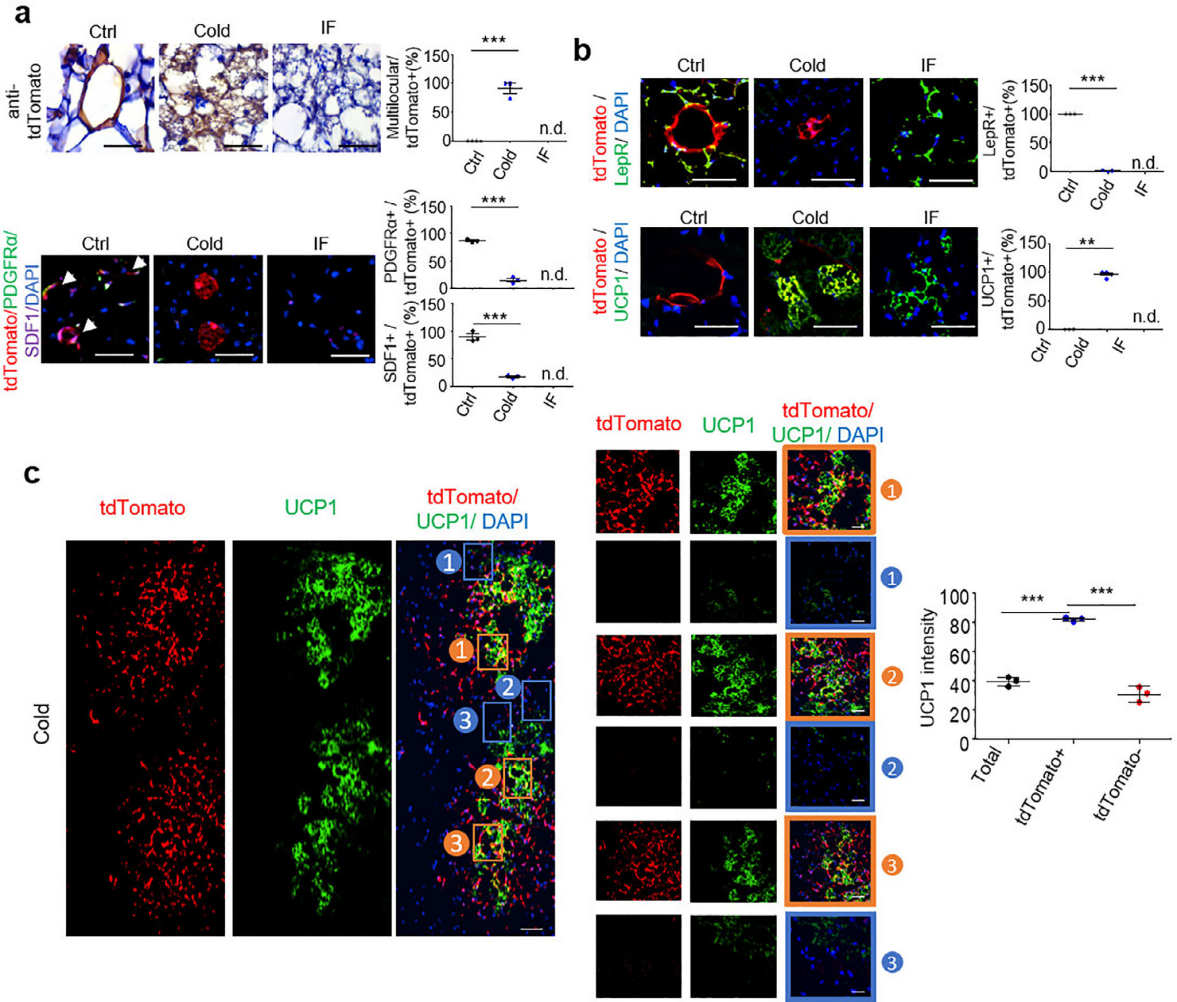
Characterization of LepR‐lineage cells during cold exposure and intermittent fasting. (a) Upper, anti‐tdTomato immunohistochemistry and quantification of tdTomato‐positive cells with multilocular morphology. Lower, tdTomato, PDGFRα, SDF1/CXCL12, and DAPI immunofluorescence; the percentage of PDGFRα‐positive cells among tdTomato‐positive cells was quantified. (b) tdTomato with LepR or UCP1 in iWAT and corresponding within‐lineage quantification. (c) UCP1 staining in cold‐exposed iWAT with quantification of UCP1 signal in tdTomato‐positive and tdTomato‐negative cells. Each plotted point represents one mouse. Data are mean ± SEM. One‐way ANOVA with Tukey's multiple‐comparison test was used unless otherwise indicated. ^*^ p < 0.05, ^**^ p < 0.01, ^***^ p < 0.001. Scale bars, 50 µm.

### 2.3 LepR‐Lineage Cells Contribute to Cold‐induced WAT Browning

To elucidate the role of LepR‐lineage cells in WAT browning, we first traced the fate of tdTomato‐labeled LepR‐lineage cells in *LepR‐Cre/tdTomato* mice with or without cold exposure or IF [26]. Immunohistochemistry (Figure 3a) and immunofluorescence (Figure 3b) showed that LepR^+^ cells were predominantly localized within the interstitial spaces between adipocytes. In the control group, LepR^+^ cells were observed among large unilocular adipocytes, whereas under cold exposure they were detected among smaller adipocytes with morphological features consistent with browning. In contrast, LepR^+^ cells were markedly reduced in the IF group (Figure 3a,b). Co‐immunofluorescence further showed that most tdTomato^+^ cells in control iWAT expressed PDGFRα, with SDF1/CXCL12 providing additional support for their stromal identity. PDGFRα expression decreased after cold exposure, consistent with differentiation of the LepR‐lineage stromal/progenitor population (Figure 3a). Notably, the LepR+ cells in the control group were unilocular, whereas those in the cold group were multilocular (Figure 3b). In the control group, LepR+ cells were positive for LepR but negative for UCP1 and other beige markers, whereas the LepR+ cells in the cold group lost LepR expression and showed increased UCP1, CD137, TMEM26, COX4, and PGC‐1α levels (Figure 3b and Figure S1a). Interestingly, we observed an increase in tdTomato‐positive cells with multilocular morphology under cold conditions compared to controls (Figure 3a). Additionally, LepR+ cells showed a significant increase in UCP1 expression relative to LepR‐ cells in the cold group, indicating enhanced thermogenic potential (Figure 3c). Figure S1b,c represent distinct experimental time points and address different phases of the IF response. Figure S1b shows the early response after 1 and 3 days of IF, when tdTomato^+^ cells remained detectable but showed increased cleaved caspase‐3 positivity, indicating the induction of apoptosis. In contrast, Figure S1c shows mice at the end of the 1‐week IF period, when tdTomato^+^ cells were nearly undetectable, as well as mice examined after 4 weeks of refeeding, when tdTomato^+^ cells reappeared. Thus, the apparent absence of tdTomato^+^ cells in Figure S1c reflects the later time point and is consistent with the progressive loss of these cells during prolonged IF, rather than an inconsistency between the two panels. Cleaved caspase‐3‐positive signals were minimal after refeeding, indicating that apoptosis was not prominent during the recovery period (Figure S1c). These data suggest LepR+ cells exhibit different responses to cold and IF treatment.

### 2.4 Ablation of LepR‐lineage Cells Reduces Cold‐induced WAT Browning

We have also quantified the contribution of LepR+/UCP1+ cells in the overall fat pad. We initially captured wide‐field images to assess the overall distribution of fluorescence signals across the sample (Figure 4a, left panel). In the cold‐exposed group, approximately 52% of UCP1‐positive beige adipocytes were tdTomato‐positive, indicating their LepR‐lineage origin. Subsequently, high‐magnification imaging with a narrower field of view was employed to resolve finer details of cellular structures (Figure 4a, right panel). We achieved cell ablation to study in vivo cell function using LepR‐Cre‐inducible DTR transgenic mice, with depletion efficiency confirmed by tdTomato fluorescence quantification (Figure 4b and Figure S14). DT administration reduced the number of LepR‐lineage cells under both control and cold conditions. Under cold exposure, LepR‐lineage cell ablation reduced UCP1‐positive area from approximately 80% to 30% (Figure 4c). In contrast, under IF conditions, DT treatment did not produce an appreciable additional reduction in UCP1‐positive area compared with IF alone (Figure 4c). Because approximately 52% of UCP1‐positive cells were tdTomato‐positive after cold exposure, LepR‐lineage cells represent an important source of cold‐induced beige adipocytes.

**FIGURE 4:**
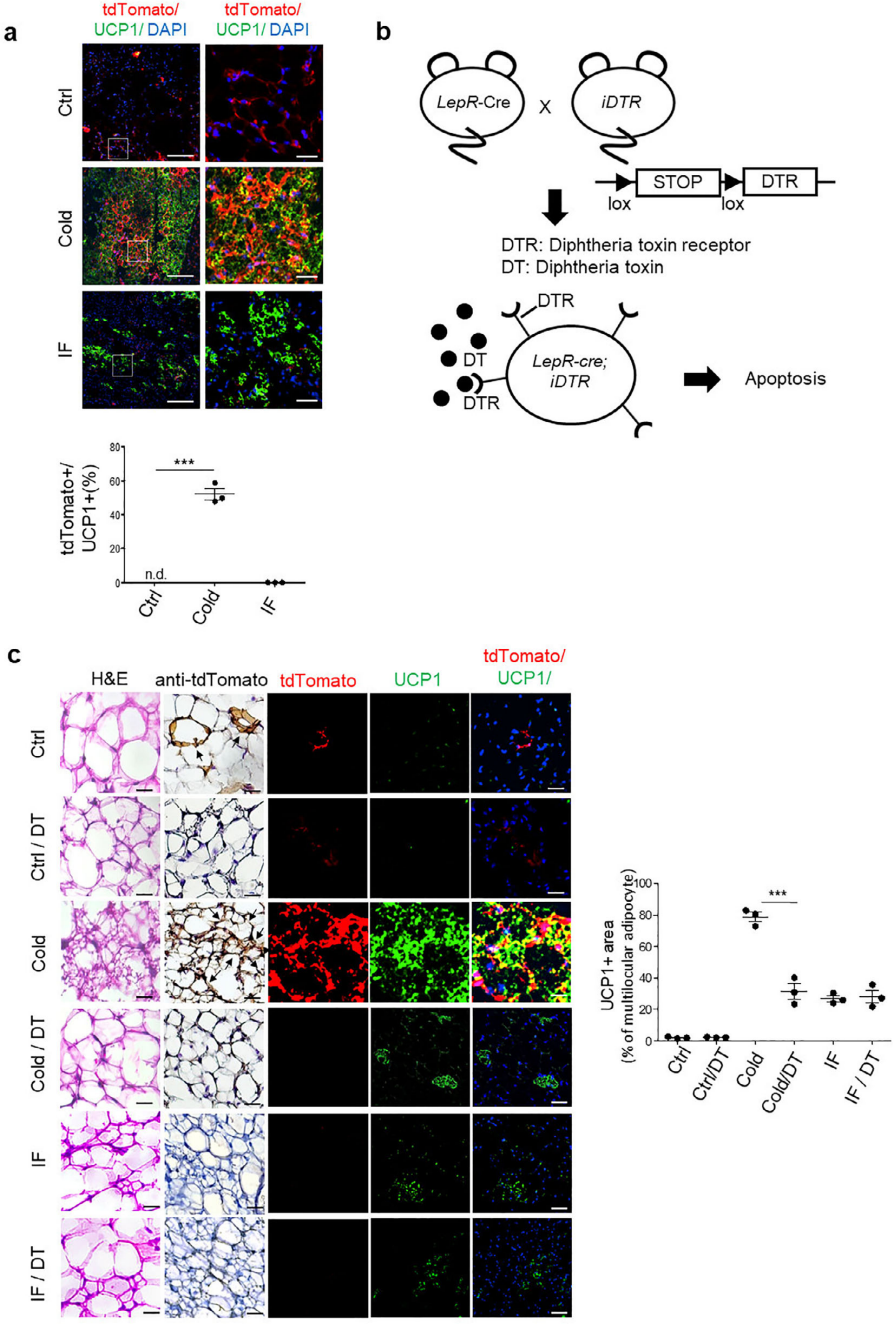
Systemic depletion of LepR‐lineage cells reduces cold‐induced WAT browning. (a) Wide‐field and higher‐magnification immunofluorescence of tdTomato and UCP1 in iWAT; approximately 52% of UCP1‐positive cells were tdTomato‐positive after cold exposure. (b) Schematic of LepR‐Cre;iDTR‐mediated depletion after diphtheria toxin (DT) administration. (c) Representative H&E staining, anti‐tdTomato immunohistochemistry, and tdTomato/UCP1 immunofluorescence in Ctrl, Ctrl/DT, Cold, Cold/DT, IF, and IF/DT groups. UCP1‐positive area was quantified using the denominator specified on the plot. Each plotted point represents one mouse. Data are mean ± SEM. One‐way ANOVA with Tukey's multiple‐comparison test was used. ^*^ *p* < 0.05, ^**^ *p* < 0.01, ^***^ *p* < 0.001. Scale bars, 50 µm.

### 2.5 Nociceptive Nerve Localization During Cold Exposure and Intermittent Fasting

Using the snRNA‐seq dataset, we first compared nuclei with detectable endogenous *Lepr* expression (*Lepr*‐positive; raw UMI > 0) with nuclei without detectable *Lepr* transcripts (*Lepr*‐negative). GSEA of the ranked differential‐expression results identified pathways related to focal adhesion, cytoskeletal regulation, integrin binding, mitochondrial fatty acid β‐oxidation, GPCR signaling, and β‐adrenergic receptor signaling (Figure 5a, left). We then asked whether the pathway‐level patterns observed in this cell‐resolved analysis were also detectable at the whole‐tissue level. Bulk RNA‐seq of whole iWAT from the same three treatment conditions used for snRNA‐seq (Control, Cold, and IF) showed corresponding treatment‐associated changes in CGRP signaling components [33]. In particular, the heatmap showed lower expression after cold exposure of *Ramp1*, *Ramp2*, *Ramp3*, *Prkca*, and *Calcrl* (Figure 5a, right). Together, these analyses identified related GPCR‐associated transcriptional patterns at the nuclear and whole‐tissue levels. The independently replicated bulk RNA‐seq findings provide complementary tissue‐level evidence (Figure S2).

**FIGURE 5:**
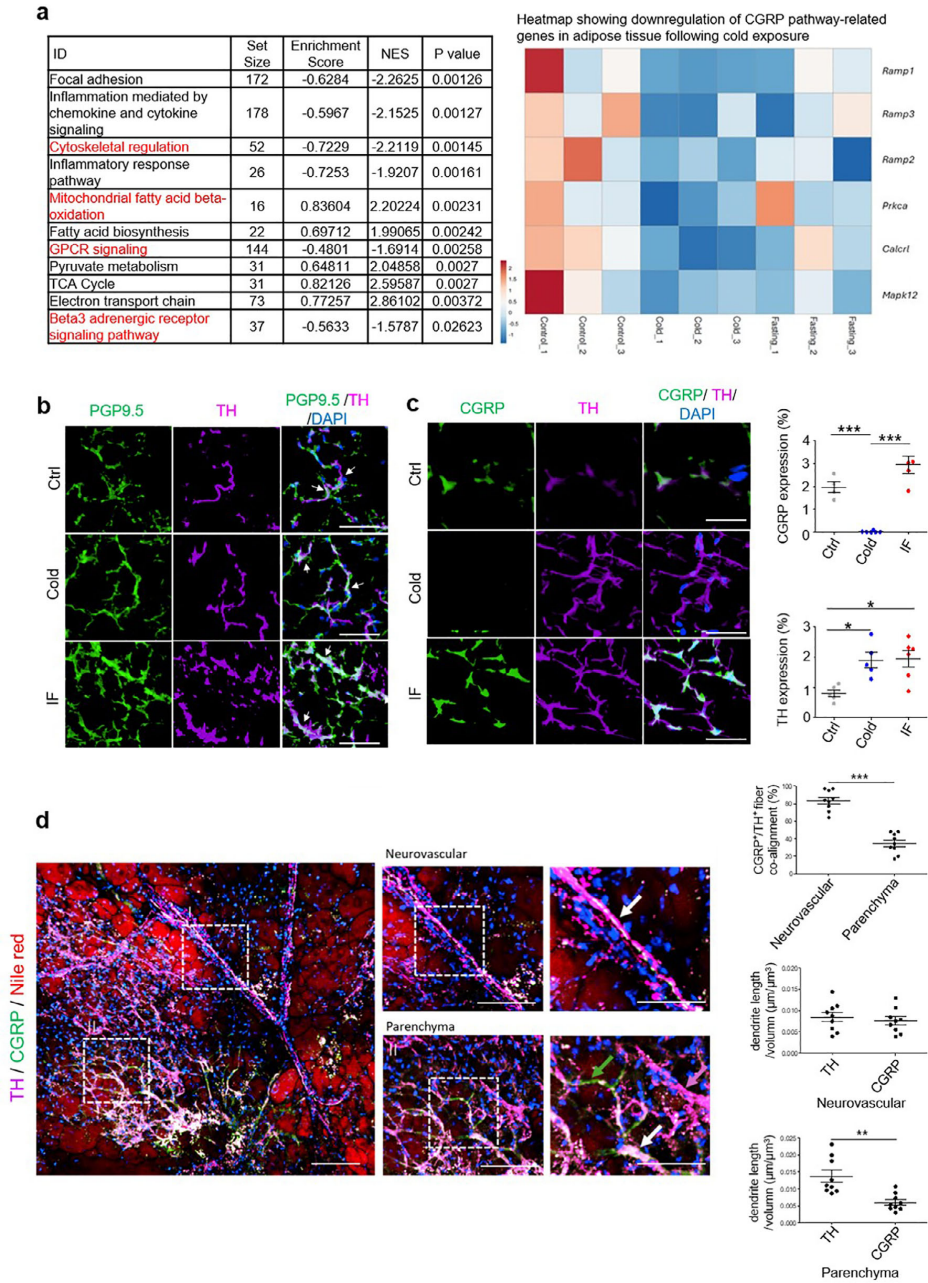
Sympathetic and CGRP‐containing sensory fibers in iWAT. (a) GSEA of genes ranked by the *Lepr*‐positive versus *Lepr*‐negative snRNA‐seq comparison (left) and a heatmap of CGRP‐receptor pathway components in whole‐iWAT bulk RNA‐seq across Control, Cold, and IF conditions (right). The bulk analysis is not cell‐type specific. (b) PGP9.5 and TH immunofluorescence. (c) TH and CGRP immunofluorescence with signal‐density quantification. (d) Three‐dimensional whole‐mount reconstructions of TH‐positive sympathetic fibers, CGRP‐positive sensory fibers, and Nile Red‐labeled adipocytes. Boxed regions are magnified. The quantified endpoint is the fraction of traced CGRP‐positive fibers aligned with TH‐positive neurovascular trajectories. Three fields were averaged per mouse; n = 3 mice. Scale bars, 200 µm.

Despite the presence of CGRP in WAT [34], WAT browning is only known to be driven by sympathetic innervation through activation of β3‐adrenergic receptor signaling pathways [6]. The role of nociceptive nerves, particularly under conditions such as cold exposure or IF, remains largely unclear. Therefore, we sought to elucidate the cellular basis of the nociceptive nerve‐WAT interaction, which might account for the association between nociceptive‐fiber CGRP immunoreactivity and inguinal WAT browning. TH+ sympathetic and CGRP+ sensory fibers have previously been described as distinct neuronal populations that coexist within adipose neurovascular bundles [35], whereas some reports have suggested direct TH/CGRP co‐expression [36, 37]. To clarify this issue, we examined the spatial relationship between TH+ sympathetic and CGRP+ sensory fibers in inguinal WAT. TH+ fibers co‐expressed the pan‐neuronal marker PGP9.5, confirming their neuronal identity (Figure 5b). Conventional confocal microscopy initially suggested apparent TH/CGRP colocalization surrounding CD31+ vessels within neurovascular bundles (Figure 5c and Figure S3a). However, super‐resolution confocal imaging demonstrated that TH and CGRP were not truly co‐expressed within the same fibers (Figure S3c). Instead, TH+ and CGRP+ fibers were closely co‐aligned within shared neurovascular bundles. Together, these findings indicate that CGRP is localized to sensory neural tracts that run adjacent to, but are distinct from, TH+ sympathetic fibers.

TH‐positive fiber density increased under cold exposure and IF. CGRP immunoreactivity was nearly absent after cold exposure, whereas IF showed a non‐significant trend toward an increase (Figure 5c). Whole‐mount imaging further showed TH‐positive and CGRP‐positive fibers traveling along shared neurovascular trajectories, with less association in parenchymal regions (Figure 5d and Figure S3a,b). These findings support a structural framework in which sympathetic and nociceptive fibers are closely co‐organized within neurovascular bundles to coordinate WAT browning.

### 2.6 Signaling Pathways Involved in the Response of LepR+ MSCs to CGRP

Given their afferent properties, it is possible that nociceptive neurons sense the WAT environment, transmit these signals to the central nervous system, and elicit sympathetic responses via efferent fibers. Alternatively, nociceptors may exert efferent effects and directly alter organ physiology [38]. Systemic treatment of mice with resiniferatoxin (RTX) to enhance nociceptive nerve impulses increased CGRP immunoreactivity but did not alter TH (Figure 6a). RTX treatment consistently decreased UCP1 (Figure 6b), CD137, TMEM26, and mitochondrial COX4 (Figure S3d), whereas NE remained unchanged (Figure 6c). RTX treatment also increased body weight and reduced rectal temperature in the cold and IF groups (Figure S4a). We then explored the possibility that nociceptor‐associated CGRP might directly affect WAT browning by local injection of CGRP into inguinal WAT. Local administration of CGRP decreased WAT browning during cold exposure (Figure 6d). Additionally, it resulted in weight gain and decreased rectal temperature (Figure S4b,f). These tissue‐level and physiological findings support changes in the browning phenotype but do not directly establish corresponding changes in whole‐body energy expenditure or substrate utilization. Conversely, only the injection of the small‐molecule CGRP receptor antagonist BIBN4096 [39] induced WAT browning and elevated the expression of UCP1 in the control group (Figure 6e). This injection also led to weight loss and increased rectal temperature (Figure S4c). As expected, similar changes caused by treatment with RTX, CGRP, or BIBN4096 were observed in LepR+ cells (Figure S5a–c).

**FIGURE 6:**
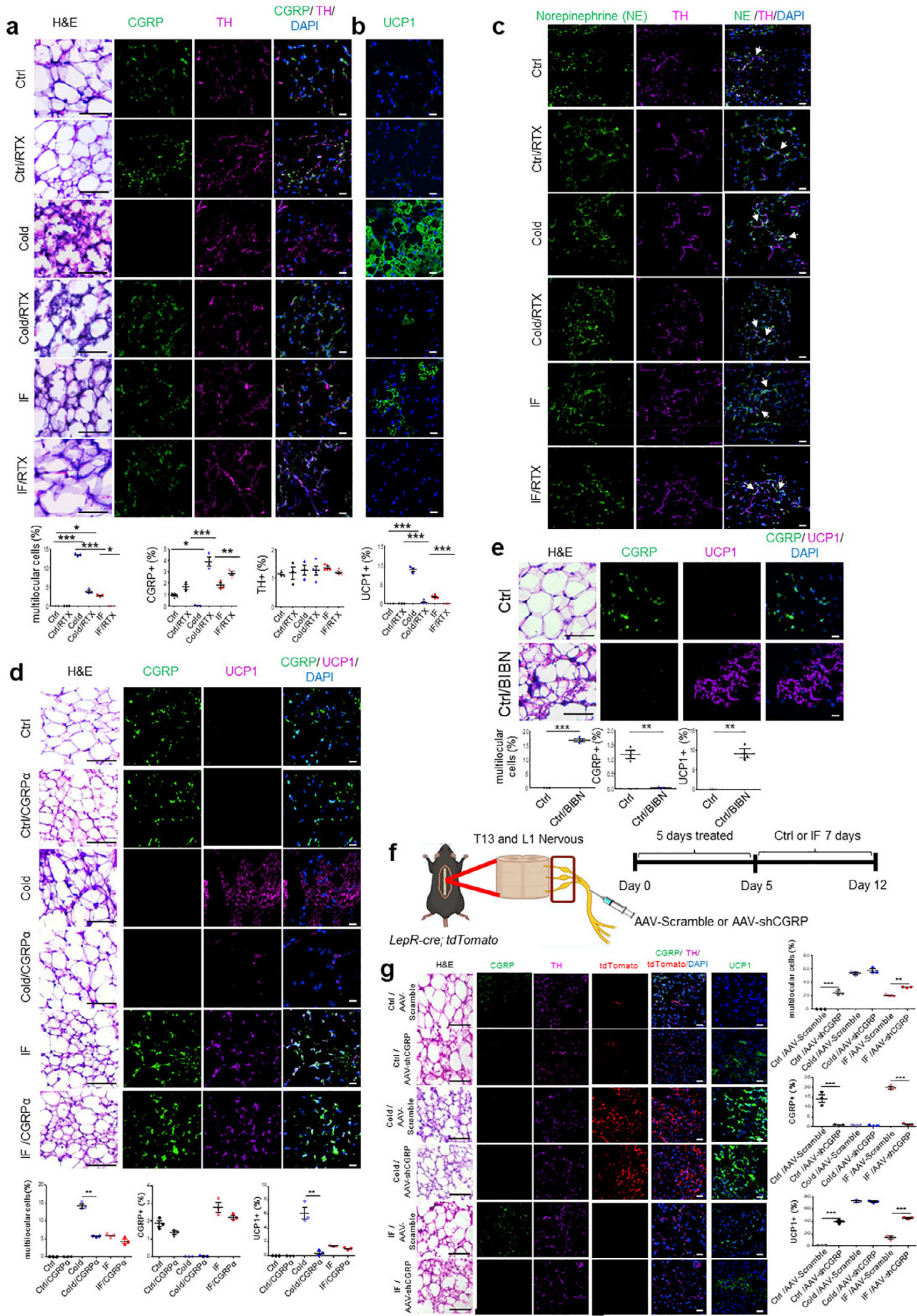
CGRP‐associated signaling modifies cold‐ and IF‐associated WAT browning. (a, b) Representative H&E staining and immunofluorescence of CGRP, TH, and UCP1 in Ctrl, Ctrl/RTX, Cold, Cold/RTX, IF, and IF/RTX groups, with quantification of multilocular adipocytes and marker signals. (c) TH and norepinephrine immunofluorescence in the same groups. (d) H&E staining and CGRP/UCP1 immunofluorescence after local CGRP administration under Ctrl, Cold, and IF conditions. (e) H&E staining and CGRP/UCP1 immunofluorescence in Ctrl and Ctrl/BIBN4096 groups. (f) Schematic of AAV9 vectors encoding CGRPα‐targeting shRNA or scrambled control injected into the T13 and L1 dorsal root ganglia. (g) H&E staining and CGRP, TH, and UCP1 immunofluorescence in AAV‐Scramble and AAV‐shCGRPα groups under Ctrl, Cold, and IF conditions. Under Cold, WAT browning did not differ between AAV‐shCGRPα and scramble groups. Each plotted point represents one mouse. Data are mean ± SEM. One‐way ANOVA with Tukey's multiple‐comparison test was used. ^*^ *p* < 0.05, ^**^ *p* < 0.01, ^***^ *p* < 0.001. Scale bars, 50 µm.

We administered AAV expressing shCGRP into the thoracolumbar DRG (T13 and L1 vertebral levels) to specifically target the nerve fibers in these ganglia (Figure 6f). AAV‐mediated CGRPα knockdown promoted beige adipocyte formation under control and IF conditions, as evidenced by H&E and UCP1 staining (Figure 6g). By contrast, under cold exposure, WAT browning did not differ between the AAV‐shCGRPα and scramble groups, possibly because the strong cold‐induced thermogenic response masked any additional effect of CGRPα depletion. AAV‐mediated CGRPα knockdown (AAV‐shCGRPα) decreased body weight in IF‐treated mice (Figure S4g). Together, these findings suggest that nociceptive CGRPα restrains WAT browning under basal and IF conditions, whereas its contribution becomes less apparent during robust cold‐induced thermogenic activation.

### 2.7 CGRP Attenuates IBMX‐induced PKA and CREB Signaling in Bone Marrow‐Derived LepR+ MSCs

Consistent with our previous observations that CGRP inhibits beige cell differentiation and reduces thermogenic capacity, we further validated this effect using in vitro murine bone marrow–derived LepR+ MSCs. Activation of protein kinase A (PKA) by 3‐isobutyl‐1‐methylxanthine (IBMX) in primary murine bone marrow LepR+ MSCs led to increased adipogenic differentiation, Oil Red O staining, PKA activity, phosphorylated cAMP response element‐binding protein (CREB), and UCP1 levels (Figure S6), suggesting that the PKA/CREB pathway is important for UCP1 expression in LepR‐MSCs [40]. CGRP co‐treatment during IBMX treatment of LepR‐MSCs did not impede adipogenic differentiation or affect Oil Red O staining (Figure S6a,b), indicating that CGRP did not broadly prevent neutral‐lipid accumulation. However, CGRP significantly attenuated IBMX‐induced PKA activation (Figure S6c) and reduced pCREB. Although UCP1 showed a decreasing trend following CGRP treatment, the difference between the IBMX and IBMX‐plus‐CGRP groups did not reach statistical significance (Figure S6d). This may reflect the strong and sustained cAMP‐elevating effect of IBMX, which could partially override CGRP‐mediated inhibition of proximal PKA/CREB signaling and preserve downstream UCP1 expression. To assess whether CGRPα influences cell growth, we found that CGRPα at concentrations of 10^−^^8^ to 10^−^^6^ M suppressed proliferation (Figure S6e) and induced apoptosis at these concentrations (Figure S6f). Together, these findings indicate that CGRP attenuates PKA/CREB signaling in vitro, while its inhibitory effect on UCP1 protein expression under strong IBMX stimulation remains inconclusive. When considered together with our in vivo findings, the results support a context‐dependent inhibitory role for nociceptor‐derived CGRP in WAT browning. Although CGRP reduced CREB phosphorylation and UCP1 expression at day 5, most remaining cells retained viability by Calcein‐AM/EthD‐1 staining (Figure S6g). In addition, Oil Red O staining at day 14 showed preserved neutral lipid accumulation, suggesting that CGRP preferentially suppresses the thermogenic program rather than globally blocking adipogenic differentiation.

### 2.8 Local TRPM8 and TRPV1 Antagonism Is Associated With Opposing Changes in CGRP Immunoreactivity and WAT Browning

To broadly examine TRP‐family expression, we performed an exploratory RT‐qPCR survey of TRP‐family transcripts in whole iWAT. This analysis revealed broad condition‐dependent changes across multiple TRP subfamilies (Figure S7). Notably, whole‐iWAT RT‐qPCR and immunofluorescence analyses showed distinct patterns of TRPM8 and TRPV1 expression. This discrepancy may reflect differences in the biological compartments assessed, as whole‐tissue RT‐qPCR measures transcripts from mixed cell populations, whereas immunofluorescence provides spatial information on protein localization, including signals from sensory nerve fibers. We next examined TRPM8 and TRPV1 protein expression in iWAT under Control, Cold, and IF conditions (Figure 7a). Local AMG333 administration [41] increased CGRP immunoreactivity and was associated with reduced cold‐induced inguinal WAT browning, as evidenced by decreased multilocular adipocyte morphology and UCP1 expression (Figure 7b), increased body weight, and reduced rectal temperature (Figure S4b). Conversely, AMG9810 [42] reduced CGRP immunoreactivity and was associated with enhanced IF‐associated WAT browning, as evidenced by increased multilocular morphology and UCP1 expression (Figure 7c), reduced body weight, and increased rectal temperature (Figure S4d). These tissue‐level and physiological findings support changes in the browning phenotype but do not directly establish corresponding changes in whole‐body energy expenditure or substrate utilization. We performed co‐staining with the pan‐neuronal marker PGP9.5 to determine whether TRPM8 and TRPV1 were localized to nerve fibers. TRPM8 and TRPV1 colocalized with PGP9.5, supporting their neuronal localization within inguinal WAT, although this analysis does not identify the specific neuronal subtypes involved (Figure 7d). Corresponding changes were detected in LepR^+^ cells following AMG333 or AMG9810 treatment (Figure S5b,d). Taken together, these local pharmacological findings suggest that TRPM8 and TRPV1 may exert opposing influences on CGRP‐associated regulation of WAT browning. TRPM8 inhibition was associated with increased CGRP immunoreactivity and reduced cold‐induced browning, whereas TRPV1 inhibition was associated with decreased CGRP immunoreactivity and enhanced IF‐induced browning. However, because CGRP secretion was not measured directly and global *Trpm8*‐ or *Trpv1*‐deficient mice did not reproduce the corresponding pharmacological phenotypes (data not shown), these findings should be interpreted as evidence of an association rather than definitive proof of a TRPM8/TRPV1–CGRP causal pathway.

**FIGURE 7:**
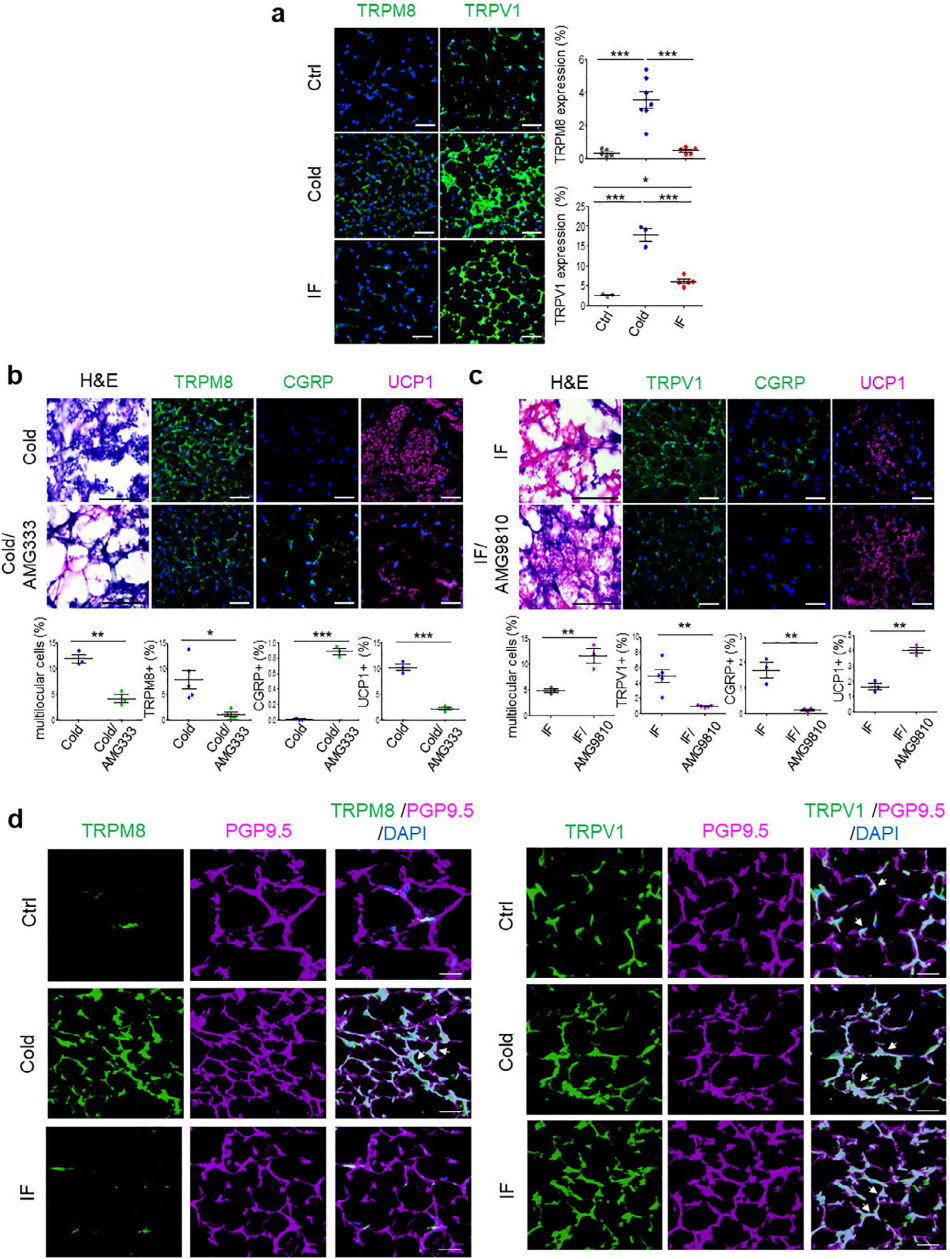
TRPM8 and TRPV1 expression and local pharmacological antagonism are associated with WAT browning. (a) Representative immunofluorescence images and quantification of TRPM8 and TRPV1 in inguinal WAT from Ctrl, Cold, and IF mice. (b) Representative H&E staining and immunofluorescence images of TRPM8, CGRP, and UCP1 in inguinal WAT from Cold and Cold/AMG333 mice, with quantification. (c) Representative H&E staining and immunofluorescence images of TRPV1, CGRP, and UCP1 in inguinal WAT from IF and IF/AMG9810 mice, with quantification. (d) Representative immunofluorescence images of TRPM8 or TRPV1 with the pan‐neuronal marker PGP9.5 in inguinal WAT from Ctrl, Cold, and IF mice. Data are mean ± SEM. Differences in (a) were analyzed by one‐way ANOVA with Tukey's multiple‐comparison test; comparisons in (b) and (c) were analyzed using two‐tailed paired Student's t‐tests. ^*^ *p* < 0.05, ^**^ *p* < 0.01, ^***^ *p* < 0.001. Scale bars, 50 µm.

### 2.9 Localization of CGRP Receptor Components in LepR‐Lineage Cells

The functional CGRP receptor is composed of three proteins: a G‐protein‐coupled receptor known as the calcitonin receptor‐like receptor (CRLR), a single transmembrane domain protein called receptor activity‐modifying protein 1 (RAMP1), and an intracellular protein, receptor component protein (RCP), whose localization has been revealed in central neurons, such as the cerebellum [43], while the detailed localization in inguinal WAT remains unclear. Immunofluorescence was performed to provide insight into CRLR, RAMP1, and RCP expression in WAT and their colocalization with LepR+ cells. CRLR, RAMP1, and RCP colocalized with LepR in the control group (Figure S8a,b), suggesting that LepR+ cells were positive for the three CGRP receptor proteins. Notably, in the cold group, a reduction in all three proteins was observed in LepR+ cells. This observation suggests that the expression of these proteins is subject to positive feedback control via interactions between ligands and receptors. Furthermore, the reduced CGRP immunoreactivity during cold exposure was likely responsible for the subsequent decrease in the expression of these proteins.

Since LepR‐MSCs with different differentiation commitments are located differently in the mouse bone marrow, with osteogenic cells closer to the arterioles and adipogenic cells closer to the sinusoids [28], we examined the location of LepR+ cells in inguinal WAT. Interestingly, LepR+/CGRP receptor+ cells were closely associated with endomucin (EMCN)‐high sinusoids but not with Sca‐1+ arterioles (Figure S8c). Notably, the density of EMCN‐high sinusoids was much higher in the cold group than in the control and IF groups (Figure S8c), which is supported by previous findings that brown/beige adipocyte‐secreted growth factors, such as BMP8b, are important for both neurovascular remodeling and energy dissipation [44].

### 2.10 CGRP‐Associated Gαi Signaling and the PKA‐CREB Pathway

During cold exposure or intermittent fasting (IF), noradrenaline released by the sympathetic nervous system binds to the β3‐adrenergic receptors and induces WAT browning [6]. In contrast, CGRP, a neuropeptide involved in nociceptive pain, acts on the G protein‐coupled receptor (GPCR), the largest family of transmembrane signaling molecules. These receptors are linked to one or more of the three major G protein families: Gs, Gi, and Gq [45]. During the inactive or resting state, GPCRs, such as the CGRP receptor, exist in a complex with heterotrimeric Gαβγ proteins. However, upon ligand binding and activation, the Gα subunit separates from the Gβγ subunit. This dissociation triggers a conformational change in the Gα subunit, leading to the exchange of guanosine diphosphate (GDP) for guanosine triphosphate (GTP). The GTP‐bound Gα subunit then initiates downstream signaling cascades, transmitting the cellular response to the ligand‐induced activation of GPCR. Gs proteins activate adenylyl cyclase (AC), increasing cAMP production, whereas Gi/o proteins inhibit it [46]. Mouse adipocytes express numerous Gi‐coupled receptors and mice lacking functional Gαi proteins exhibit impaired whole‐body glucose homeostasis and insulin sensitivity [47]. These findings suggest a negative role for CGRP‐Gi signaling in regulating the cAMP/PKA/CREB pathway (Figure 8a).

**FIGURE 8:**
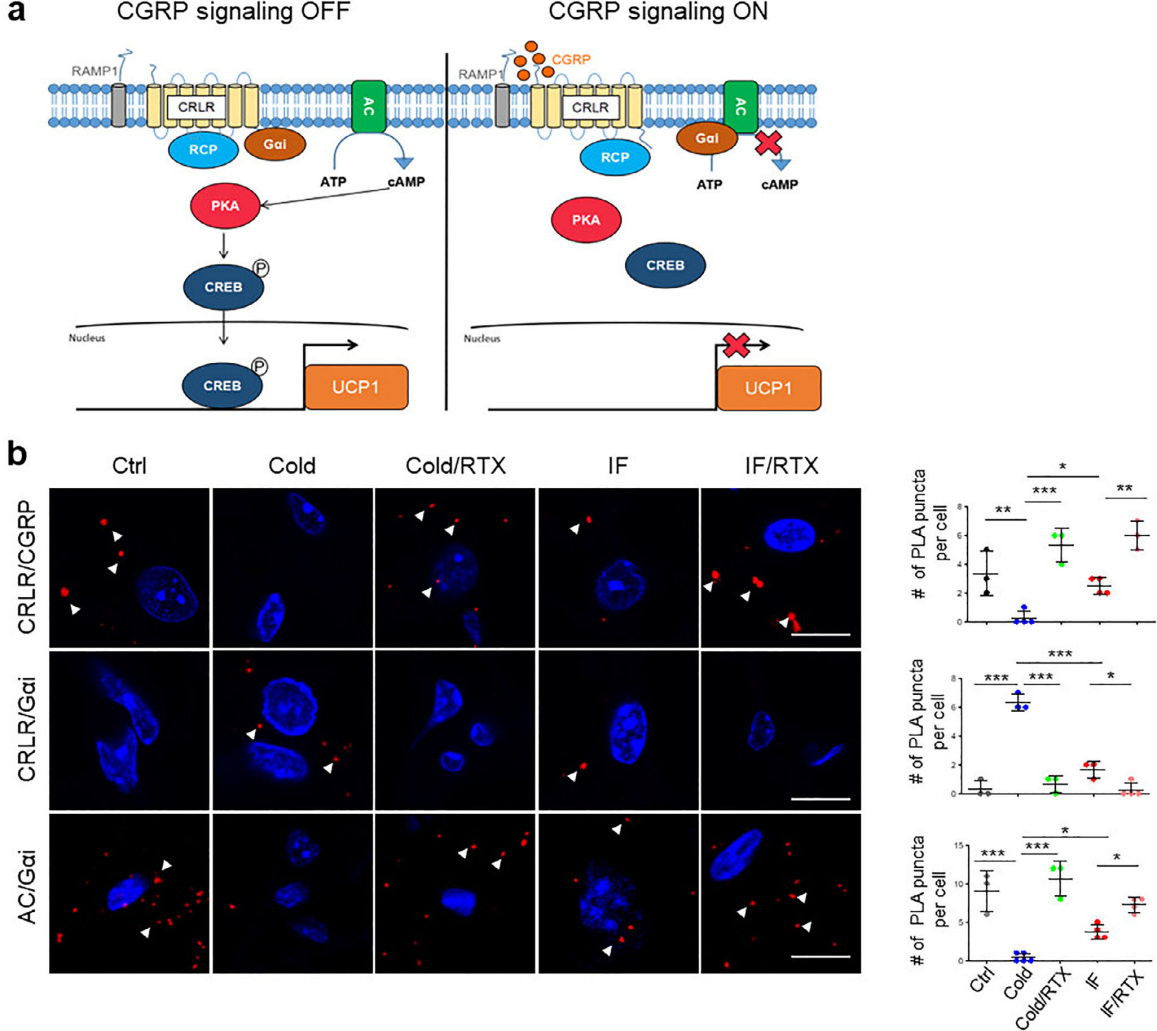
Proposed CGRP‐Gαi model and molecular‐proximity analysis. (a) Proposed model in which CGRP binding to CRLR engages Gαi and is associated with reduced AC‐dependent cAMP production, PKA activity, CREB phosphorylation, and UCP1 expression. (b) PLA analysis of CRLR/CGRP, CRLR/Gαi, and AC/Gαi proximity in iWAT from Ctrl, Cold, Cold/RTX, IF, and IF/RTX mice, with quantification. Each plotted point represents one mouse. Data are mean ± SEM. One‐way ANOVA with Tukey's multiple‐comparison test was used. ^*^ *p* < 0.05, ^**^ *p* < 0.01, ^***^ *p* < 0.001. Scale bars, 10 µm.

Double immunofluorescence (Figure 8b) and Duolink Proximity Ligation Assay (PLA) densities of CRLR/CGRP and AC/Gαi were higher in both the control and IF groups than in the cold group, whereas PLA signal densities of CRLR/Gi were higher in the cold group than in the control and IF groups (Figure 8b). RTX treatment was found to increase PLA signal densities of CRLR/CGRP and AC/Gαi while decreasing PLA signal densities of CRLR/Gi in inguinal WAT in both the cold and IF groups (Figure 8b and Figure S9a). These results were consistent with RTX‐associated changes in CGRP immunoreactivity. In the cold group, the injection of CGRP into the inguinal WAT resulted in the proximity of CGRP and CRLR. This was accompanied by altered proximity of Gαi to CRLR and AC, as evidenced by the PLA signal densities of CRLR/CGRP and AC/Gαi and the decreased PLA signal densities of CRLR/Gαi (Figure S9b). Previous studies have shown that subcutaneous white adipose tissue expresses higher levels of α2‐adrenergic receptors, which are coupled to Gαi [48, 49]. We have now provided evidence that LepR+ cells colocalize with Gαi, rather than Gαs, under control conditions where CGRP is detectable (Figure S9c). These findings are consistent with involvement of a CGRP‐Gαi‐CREB signaling axis, although PLA demonstrates molecular proximity rather than direct binding or pathway activation. To obtain functional support, we used a selective Gαi inhibitor (0990CL) [50]. Gαi blockade significantly enhanced pCREB and UCP1 signaling in both control and IF‐treated mice. Quantitative analysis revealed a significant increase in the number of pCREB+ nuclei (Figure S10) and a corresponding increase in UCP1+ area (Figure S10) following Gαi inhibition compared with control or IF alone. Under cold exposure, however, pCREB and UCP1 were already strongly elevated, and Gαi inhibition produced no significant additional increase. Cold‐induced sympathetic and β‐adrenergic signaling activates CREB and promotes UCP1 expression; therefore, the incremental effect of Gαi inhibition may have been limited under this condition. This may reflect a ceiling effect or reduced dynamic range, although cAMP levels and pathway saturation were not directly assessed. Together, these findings support a model in which CGRP–CRLR signaling engages Gαi to suppress downstream PKA–CREB activity.

### 2.11 Macrophage‐associated Clearance of LepR‐Lineage Cells During Intermittent Fasting

We proceeded to investigate the underlying mechanisms driving apoptosis and subsequent loss of LepR+ cells in the IF group. Exploratory snRNA‐seq analysis (Figure 9a) identified a greater representation of nuclei annotated with macrophage‐ and dendritic‐cell‐associated signatures in the IF dataset than in the Control and Cold datasets. Given the pivotal role of macrophages in cell efferocytosis and their characteristic formation of crown‐like structures (CLS) around targeted cells [51], we further examined macrophage infiltration through double immunofluorescence. Over the course of IF, the numbers of F4/80^+^ macrophages and CLS progressively increased relative to day 0. Higher‐resolution confocal imaging with orthogonal (xy, xz, and yz) views further revealed tdTomato‐positive LepR‐lineage cellular material within F4/80‐positive macrophages in CLS, providing more direct evidence consistent with macrophage engulfment of LepR‐lineage cells (Figure 9b). Notably, depletion of macrophages via pretreatment with clodronate liposomes in IF group mice resulted in decreased F4/80+ macrophage numbers and CLS and the reappearance of LepR+ cells (Figure 9c), accompanied by a reduction in body weight and an increase in rectal temperature (Figure S4e). Immunofluorescence further revealed that F4/80+ macrophages are positive for CGRP receptor and co‐receptor, CRLR, RAMP1, and RCP, on the third day of IF (Figure 9d). Interestingly, only the injection of the small‐molecule CGRP receptor antagonist BIBN4096 led to a reduction in the number of infiltrating F4/80+ macrophages and CLS, while the injection of the CGRP agonist did not alter these numbers (Figure 9e). Importantly, LepR^+^ cells that became nearly undetectable after one week of IF were not restored by CGRPα knockdown in the targeted thoracolumbar DRG (Figure 6g), whereas BIBN4096 treatment promoted their reappearance during IF (Figure 9e). These distinct outcomes may reflect the different levels at which the two interventions disrupt the pathway. DRG‐directed AAV‐shCGRPα reduces CGRPα production in selected sensory neurons but may not eliminate residual input from incompletely transduced fibers, adjacent ganglia, other neuronal populations, or related ligands. By contrast, BIBN4096 blocks CGRP receptor signaling at the recipient‐cell level and may therefore suppress convergent CGRP‐related inputs more comprehensively. We speculate that receptor blockade releases a signaling constraint that permits residual or quiescent LepR‐lineage cells to survive, expand, or restore detectable LepR expression during prolonged IF. Accordingly, the reappearance of LepR^+^ cells following BIBN4096 treatment may reflect receptor‐level restoration of the LepR^+^ stromal‐cell compartment rather than merely recapitulating the effect of reduced neuronal CGRPα production. The reappearing LepR^+^ cells showed heightened UCP1 expression (Figure 9f).

**FIGURE 9:**
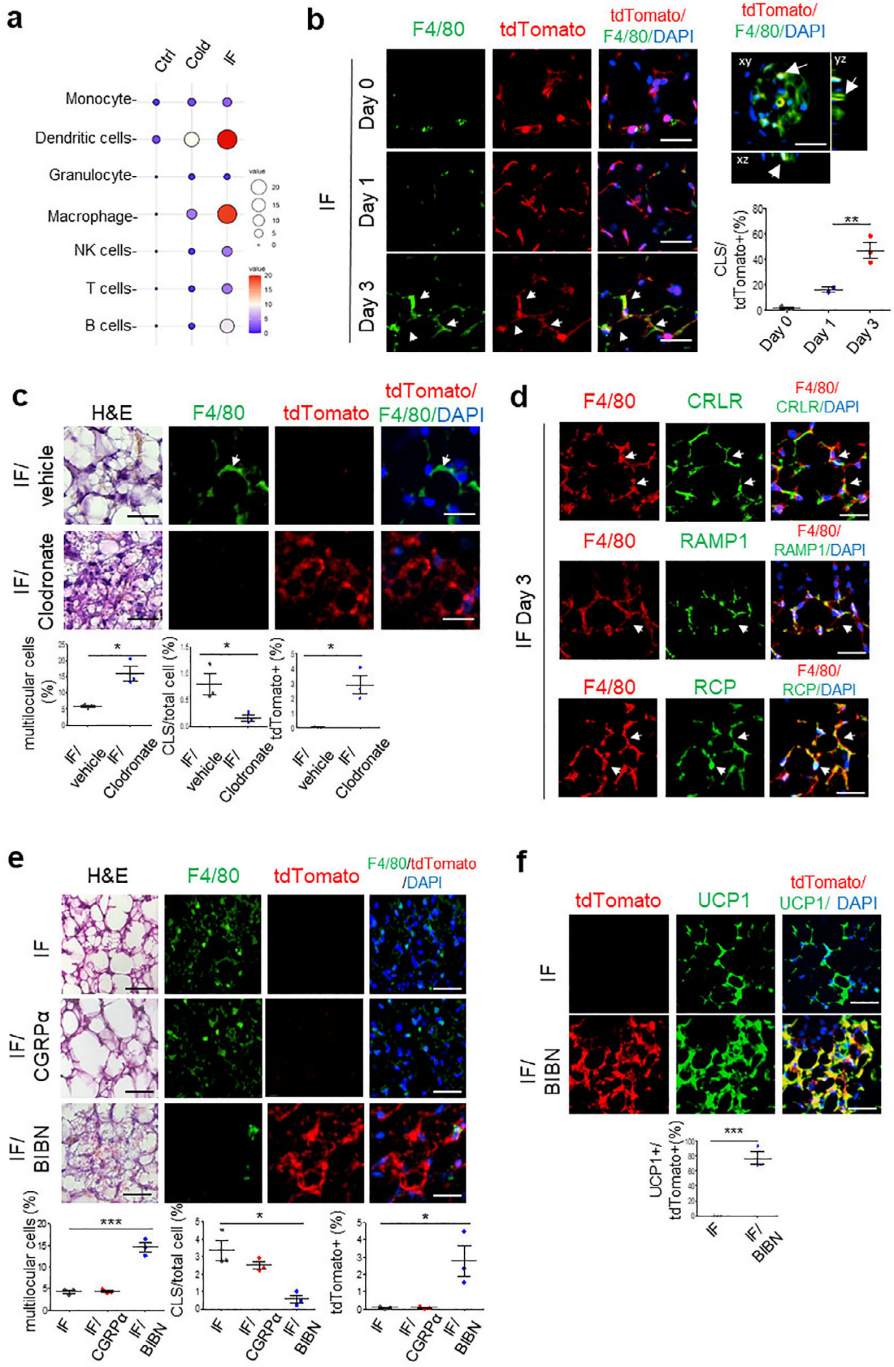
Macrophage‐associated clearance of LepR‐lineage cells during intermittent fasting. (a) Transcriptomic analysis of macrophage‐ and dendritic‐cell‐associated signatures in iWAT from the Control, Cold, and IF groups. Dot size indicates the fraction of cells expressing each marker, and color indicates average expression level. (b) F4/80 and tdTomato immunofluorescence on IF day 0 and day 3. Arrows indicate crown‐like structures (CLS); orthogonal xy, xz, and yz views show tdTomato‐positive material within F4/80‐positive macrophage profiles. (c) H&E staining and F4/80/tdTomato immunofluorescence in IF/vehicle and IF/clodronate groups. (d) CRLR, RAMP1, and RCP immunofluorescence in F4/80‐positive macrophages. (e) H&E staining and F4/80/tdTomato immunofluorescence in IF, IF/CGRPα, and IF/BIBN4096 groups. (f) UCP1 and tdTomato immunofluorescence in IF/vehicle and IF/BIBN4096 groups. The percentage of tdTomato‐positive cells that were UCP1‐positive was calculated only when a tdTomato‐positive denominator was detectable. Each plotted point represents one mouse. Data are mean ± SEM. Two‐group comparisons used two‐tailed unpaired Student's t tests; multi‐group comparisons used one‐way ANOVA with Tukey's test. ^*^ p < 0.05, ^**^ p < 0.01, ^*^ ^**^ p < 0.001. Scale bars, 50 µm.

### 2.12 Human Adipose Single‐Cell Datasets Identify a Marker‐Defined CGRP‐Receptor‐Associated Population

To investigate whether a similar cell population is present in human adipose tissues, we performed single‐cell RNA‐seq analysis of both human white adipose [32] and brown adipose tissues [52]. Our analysis revealed the existence of one cluster of cells (hEndoS3) that expressed *LEPR*, *CALCRL* (encoding CRLR), *CRCP* (encoding RCP) and *RAMP3* (encoding RAMP3) in human subcutaneous adipose tissue; however, the same cell clusters showed decreased mRNA levels of some molecules in human visceral adipose tissue (Figure S11a–c). Notably, adrenomedullin 2, a member of the CGRP family, selectively binds to CRLR/RAMP3 rather than to CRLR/RAMP1 or CRLR/RAMP2 in the adipose tissues of humans [53]. These data identify a human stromal population with partial marker correspondence to mouse LepR‐lineage cells but do not establish functional equivalence or CGRP responsiveness. However, in human BAT, we identified a cluster of cells in the adipocyte subpopulation that expressed *LEPR* and *CALCRL* but not *CRCP* and all *RAMP* genes (Figure S11d). Consistent with previous studies in mice, sensory ablation affected brown/beige molecular programs in inguinal WAT but not in interscapular BAT [14]. Taken together, these findings identify partial marker correspondence between mouse and human adipose populations and highlight the need for functional studies using human‐derived beige adipocyte progenitors.

## 3 Discussion

These findings reveal an adipocyte precursor that is controlled by nociceptive CGRP and responds differently to WAT browning induced by different conditions. Higher nociceptive CGRP immunoreactivity was associated with reduced IF‐induced WAT browning, whereas exogenous CGRP suppressed cold‐induced WAT browning. LepR‐Cre lineage tracing identified LepR‐lineage cells in inguinal WAT that contribute to beige adipocyte formation and respond differently to cold exposure and IF. This difference was associated with peripheral CGRP immunoreactivity and loss of LepR‐lineage cells during IF, whereas CGRP immunoreactivity was reduced during cold exposure. Local pharmacological experiments further suggested that CGRP‐associated signaling is influenced by TRPV1 and TRPM8. These associations require cell‐specific genetic validation and direct measurement of CGRP secretion. The delayed rectal‐temperature response during IF may reflect progressive adipose remodeling and adaptation to repeated fasting–feeding cycles, consistent with previous observations of iWAT beiging under alternate‐day fasting [29].

We focused on subcutaneous inguinal WAT because cold exposure increases the thermogenic capacity of BAT and browning of inguinal WAT, with less extensive browning of visceral perigonadal WAT [54]. Inguinal WAT is also more densely innervated by thoracolumbar DRG nerves than epididymal or periovarian WAT [14]. Lineage tracing in LepR‐Cre/tdTomato mice showed that endogenous LepR‐lineage cells contribute substantially to inguinal beige adipocytes during cold exposure but not during IF.

Nociceptors rapidly detect external insults and contribute to protective immune responses [55] as well as HSC maintenance [56]. TH is traditionally used as a marker of the sympathetic nerves that dominate scWAT innervation. However, up to 40% of adipose parenchymal sensory nerves are also TH‐immunopositive [14, 57], complicating a clear distinction between sensory and sympathetic fibers. This shared marker indicates greater heterogeneity of adipose innervation and the potential for neuropeptide release from both nerve types. Our findings extend the known functions of nociceptive neurons beyond hematopoiesis and immunity by linking nerve‐associated CGRP to WAT browning under cold exposure and IF. CGRP receptor components and Gαi signaling were associated with reduced LepR‐lineage cell abundance during IF, and cleaved caspase‐3 staining indicated apoptosis. These data complement reports that selective adipose sensory ablation, with sympathetic innervation preserved, enlarges the fat pad, enriches beige adipocytes, and increases body temperature under thermoneutral conditions [14].

The preserved Oil Red O staining indicates that CGRP did not broadly prevent neutral‐lipid accumulation and is therefore more consistent with attenuation of the beige/thermogenic program than with inhibition of terminal adipogenesis per se. The accompanying reduction in cell number and increase in the sub‐G1 fraction further indicate that CGRP‐associated loss of cell viability may also contribute to the UCP1 trend.

Both cold exposure and IF increased TRPV1 expression, whereas only cold exposure increased TRPM8 in inguinal WAT. Pharmacological manipulation linked these channels to opposing changes in nociceptive CGRP immunoreactivity and WAT browning. TRPM8 contributes to tail vasoconstriction, heat retention, intake behavior, and metabolic fuel selection during cooling [58]. Accordingly, *Trpm8*‐knockout mice under mild cold show greater tail heat loss and lower core temperature, followed by late‐onset obesity and glucose and lipid dysfunction [58]. Persistent daytime hyperphagia and reduced fat oxidation may contribute to this phenotype [58], whereas the TRPM8 agonist icilin increases energy expenditure and decreases body weight [59]. In our experiments, TRPV1‐associated signaling was linked to increased CGRP immunoreactivity and reduced PKA activity, whereas TRPM8‐associated signaling was linked to reduced CGRP immunoreactivity and increased PKA activity. Their opposing effects may balance anti‐ and pro‐thermogenic signaling during cold exposure. Interactions or feedback between TRPM8 and TRPV1, and convergence of their downstream pathways on PKA or other thermogenic regulators, remain to be defined. However, these interpretations are based mainly on local antagonist experiments.

Pathway analysis indicated enrichment of adrenergic signaling, whereas expression of *Adrb3* and downstream thermogenic genes was separately examined to evaluate the β3‐adrenergic thermogenic axis. In the whole‐iWAT bulk RNA‐seq dataset, *Adrb3* expression was lower in the Cold and IF groups than in the Control group (Figure S2). Because these bulk data encompass all iWAT cell types, this observation should not be interpreted as cell type‐specific downregulation in *Lepr*‐positive stromal/progenitor cells. Sustained adrenergic stimulation can induce receptor desensitization through phosphorylation, internalization, and transcriptional repression [60], and treatment‐associated changes in tissue cellular composition may also contribute to the whole‐tissue expression pattern. Thus, pathway enrichment and *Adrb3* expression represent related but analytically distinct observations, and their cellular basis requires further validation.

The link between impaired thermogenesis and metabolic diseases such as obesity and diabetes has been recognized for decades [61], but using cold exposure or IF to promote human energy expenditure is difficult. Short‐ and long‐term administration of noradrenergic agents is often associated with tachycardia and cardiovascular events [62], while progressively colder stimuli eventually evoke burning pain [63]. Our findings suggest that this pain may arise from nociceptor activation rather than circuit‐dependent increases in sympathetic neurotransmitter release. TRPM8 agonism and CGRP blockade therefore warrant experimental evaluation for their effects on CGRP immunoreactivity and receptor engagement. CGRP‐targeting antibodies are already approved for migraine [64]. Nevertheless, our male‐mouse data do not establish changes in whole‐body energy expenditure or therapeutic effects in obesity, and these implications require direct testing.

Our immunofluorescence analyses demonstrated increased macrophage accumulation and crown‐like structure formation during IF, while snRNA‐seq provided complementary descriptive information regarding macrophage‐associated transcriptional profiles. Furthermore, F4/80‐positive macrophages expressed CGRP receptor components. BIBN4096 reduced macrophage infiltration and CLS formation, contrasting with studies in bacterial meningitis and tissue injury in which CGRP inhibited macrophage recruitment or promoted apoptosis [65, 66]. This discrepancy likely reflects tissue context, macrophage heterogeneity, and the local microenvironment. In adipose tissue, CGRP may therefore contribute not only to immunosuppression but also to macrophage recruitment or survival during remodeling.

Our findings support a condition‐ and time‐dependent model. During IF, apoptosis of LepR‐lineage cells was evident by day 3 and was followed by macrophage accumulation and crown‐like structure formation. CGRP–Gαi signaling may initially suppress PKA–CREB–UCP1 activity in surviving LepR‐lineage cells; as apoptosis and macrophage‐mediated clearance progress, physical depletion of these cells likely becomes increasingly important. Expression of CRLR, RAMP1, and RCP by IF‐associated macrophages further suggests that BIBN4096 may act on both residual LepR‐lineage cells and macrophages. In contrast, cold exposure did not cause comparable loss of LepR‐lineage cells, and exogenous CGRP or RTX nevertheless suppressed cold‐induced UCP1 expression. Thus, direct CGRP‐mediated inhibition may predominate during cold exposure, whereas both early signaling inhibition and later apoptosis and clearance contribute during IF.

Several limitations should also be considered. Although WAT browning was evaluated by adipocyte morphology, UCP1 expression, body weight, and rectal temperature, whole‐body oxygen consumption, carbon dioxide production, energy expenditure, and respiratory exchange ratio were not measured. Thus, our data establish WAT browning but do not demonstrate corresponding changes in whole‐body energy expenditure or fuel preference. Moreover, body weight and rectal temperature can be influenced by food intake, activity, heat loss, and systemic thermoregulation. Indirect calorimetry accompanied by measurements of food intake and locomotor activity will be needed to define the systemic metabolic consequences of this pathway. The snRNA‐seq analysis provided descriptive and hypothesis‐generating insights into adipose cellular heterogeneity and transcriptional‐state relationships. Given that each experimental condition was represented by a single pooled library, condition‐level comparisons were interpreted as exploratory rather than definitive evidence of changes in cell abundance, gene expression, or lineage transitions. Importantly, these transcriptomic observations complement independent evidence from lineage‐tracing, immunofluorescence, functional perturbation, and biologically replicated bulk RNA‐seq experiments, collectively strengthening the biological interpretation of the study.

Finally, several model‐specific limitations should be acknowledged. Evidence for reciprocal TRPM8 and TRPV1 involvement was derived mainly from local antagonist studies, which may involve off‐target or indirect effects. Global *Trpm8*‐ and *Trpv1*‐knockout mice did not reproduce the corresponding pharmacological phenotypes (data not shown), potentially because broad deletion affects additional neuronal and non‐neuronal circuits, systemic thermoregulation, or developmental compensation. Anatomically restricted, sensory‐subtype‐specific deletion combined with selective activation, direct CGRP‐release measurements, and genetic rescue will therefore be necessary to establish channel‐, cell‐, and site‐specific causality. Additional limitations include the absence of direct intracellular cAMP measurements, the inability of PLA to distinguish molecular proximity from direct physical binding, the lack of functional validation in human tissues, and the exclusive use of male mice. Moreover, systemic DT‐mediated ablation in LepR‐Cre;iDTR mice cannot distinguish the contribution of adipose‐resident LepR‐lineage cells from that of LepR‐expressing populations in other tissues. LepR‐Cre lineage labeling has been reported in the bone marrow, brain, liver, kidney, lung, spleen, heart, and intestine [67, 68], and depletion efficiency and inflammatory changes in these extra‐adipose tissues were not comprehensively evaluated. Therefore, although DT‐mediated depletion markedly reduced LepR‐lineage cells and attenuated cold‐induced WAT browning, these findings support a contribution of LepR‐lineage cells but do not establish an exclusively adipose‐specific causal effect. Collectively, our findings support a condition‐dependent role for nociceptive CGRP signaling and LepR‐lineage cells in WAT browning, but further cell‐specific, metabolic, and human functional studies are required before their therapeutic relevance can be established.

## 4 Methods

### 4.1 Animals

#### 4.1.1 Mice Were Purchased from the Jackson Laboratory

*Rosa26‐CAG‐loxp‐stop‐loxp‐tdTomato* mice (#007914) and *Rosa26‐loxp‐stop‐loxp‐iDTR mice* (#007900) were crossed to *LepR‐Cre* mice (#008320) and maintained on a C57BL/6 background. Male mice aged two to six months were used for the experiment. To treat mice with diphtheria toxin (DT), mice were intraperitoneally injected with 100 ng of DT (Sigma‐Aldrich) for 7 consecutive days. All animal studies and care of live animals were approved and performed in accordance with the guidelines of the China Medical University Institutional Animal Care and Use Committee 2020–290, 2022–342 and 2023‐079‐2. Reporter specificity was confirmed using LepR‐Cre‐only and tdTomato‐reporter‐only controls (Figure S12).

### 4.2 Cold Exposure

Mice were housed at 6°C (cold) for 7 days in a controlled environment with free access to food and water. Mice were maintained under a 12‐h light–dark cycle with 30% humidity on a normal diet. For drug treatment experiments, mice received the drug on day 1 before cold exposure and on day 4 during cold exposure.

### 4.3 Intermittent Fasting

Mice underwent alternating 24‐h periods of free access to food and 24‐h fasting over one week. This regimen is referred to throughout as intermittent fasting (IF).

### 4.4 Nuclei Isolation

The nuclei isolation protocol was adapted from a previous publication [69]. For each experimental condition, inguinal WAT from six mice was pooled before nuclei isolation and library preparation. The pooled tissue was cut into small pieces (1 mm^3^) with scissors and rapidly transferred to a prewetted 100 µm cell strainer in a Petri dish on ice. The tissue was treated with 3 mL of ice‐cold nuclei preparation buffer [(NPB) 10 mM HEPES (15630‐080, Life Technologies) (pH 7.5), 1.5 mM MgCl_2_ (M1028, Sigma‐Aldrich), 10 mM KCl (P9541, Sigma‐Aldrich), 250 mM sucrose (S7903, Sigma‐Aldrich), and 0.1% IGEPAL CA‐630 (NP‐40) (I8896, Sigma‐Aldrich) in DEPC water] and minced through the strainer using a syringe plunger. The tissue was kept immersed in ice‐cold NPB. The minced tissue was resuspended, passed through the strainer, and collected in the Petri dish. The plunger and strainer were washed with 2 mL of NPB, which was then passed through the strainer. The homogenate was transferred to a pre‐cooled 50 mL tube on ice and centrifuged at 1,000 × g for 10 min at 4°C. After aspiration of the lipid layer, the nuclear pellet was resuspended in 100 µL of ice‐cold nuclei resuspension buffer [(NRB) 1% BSA, 2 mM MgCl_2_ (M1028, Sigma‐Aldrich), and 0.04 U RNase inhibitor (03335399001, Roche) in 1 × PBS]. Nuclei were counted using a Countess II FL Automated Cell Counter (Thermo Fisher Scientific). Approximately 12,000 nuclei from each pooled condition were submitted for library preparation using the 10x Genomics Chromium 3′ v3.1 platform, with a target recovery of approximately 10,000 nuclei. One pooled snRNA‐seq library was generated for each condition (Control, Cold, and IF), for three libraries in total. Because tissues were pooled before library preparation, the six contributing mice were not independent snRNA‐seq replicates; the library‐level sample size was n = 1 per condition.

### 4.5 Single‐Nucleus RNA Sequencing and Data Analysis

10x Genomics sequencing libraries were prepared using the 10x Chromium Single Cell 3′ v3.1 reagent kit. Nuclei solutions containing 700–1,200 nuclei/µL were loaded into a Chromium Controller. Sequencing was performed on a NovaSeq 6000 (Illumina), and alignment, demultiplexing, and initial filtering were performed with Cell Ranger according to the manufacturer's protocol. Each library was processed independently for quality control. Nuclei with fewer than 100 detected genes in raw counts or more than 15% mitochondrial UMIs in DecontX‐corrected counts were excluded. nFeature counted genes with non‐zero raw UMIs, and top‐1 fractions were calculated from DecontX‐corrected counts using scuttle::perCellQCMetrics. Nuclei were additionally excluded only when both the top‐feature fraction was ≥80% and the corrected UMI remaining after removal of that feature was <125. Potential doublets were identified independently in each library using Scrublet, and nuclei with Scrublet scores > 0.25 were removed. After expression‐concentration filtering, Scrublet excluded 47, 32, and 142 nuclei from the Control, Cold, and IF libraries, respectively. Per‐library quality‐control metrics are provided in the accompanying (Resource Data Figure 2a). After quality control and doublet removal, 3,268 nuclei were retained (Control, 1,148; Cold, 719; IF, 1,401). Genes detected in more than 10 nuclei were retained for downstream analysis. The filtered matrices were normalized and scaled using the NormalizeData and ScaleData functions in Seurat, respectively. Cell annotation integrated scCATCH predictions [31], cluster‐specific differentially expressed genes, canonical marker programs, and published adipose single‐cell atlases. Mesenchymal/stromal cells were identified by *Pdgfra*, *Dcn*, and *Lum*; adipose progenitor/preadipocyte populations by *Dpp4*, *Pi16*, *Cd34*, *Ly6a*, *Icam1*, and *Dlk1*; mature adipocytes by *Adipoq*, *Plin1*, *Fabp4*, and *Dgat2*; and beige/thermogenic adipocytes by *Ucp1*, *Cidea*, *Ppargc1a*, *Cox8b*, and *Dio2*. For trajectory analysis, scCATCH‐annotated stem/progenitor and adipocyte populations were reanalyzed independently of *Lepr* expression in the Control‐plus‐Cold and Control‐plus‐IF datasets using Monocle3 v1.4.27 [70] and scVelo v0.3.4. Monocle3 was used to infer principal graphs and pseudotime, and scVelo provided complementary RNA‐velocity directionality. A Control stem/progenitor nucleus was selected as the pseudotime root based on cell identity and RNA‐velocity direction. Endogenous *Lepr* expression was then overlaid on the inferred trajectories (*Lepr*‐positive, raw UMI > 0; *Lepr*‐negative, raw UMI = 0). Clusters and trajectories were visualized on UMAP embeddings [71].

Differential expression between each annotated cluster and all remaining nuclei was assessed using the Wilcoxon rank‐sum test, followed by Benjamini–Hochberg correction for multiple testing. Ranked cluster‐specific differentially expressed gene lists are provided in the accompanying Resource Data. For the Figure 5a analysis, *Lepr*‐positive and *Lepr*‐negative nuclei, defined by raw *Lepr* UMI counts > 0 and = 0, respectively, were compared using the same framework, and the ranked differential‐expression results were analyzed by GSEA. Although pseudobulk analyses generally aggregate counts at the independent‐sample level [72], the pooled design yielded one snRNA‐seq library per condition; therefore, condition‐level findings were interpreted as exploratory cell‐state and pathway patterns rather than replicate‐level differential inference. Pathways identified in the *Lepr*‐positive versus *Lepr*‐negative snRNA‐seq comparison were then compared with treatment‐associated patterns in the whole‐iWAT bulk RNA‐seq dataset.

### 4.6 Bulk RNA Sequencing and Data Analysis

Total RNA was extracted from whole inguinal WAT collected from control, cold‐exposed, and intermittently fasted mice using the RNeasy Kit (Qiagen) and assessed for integrity with the RNA Nano 6000 assay kit (Agilent Technologies, CA, USA). Thus, the bulk RNA‐seq experiment represented the same three treatment conditions as the snRNA‐seq experiment but measured whole‐tissue transcriptomes rather than cell‐type‐resolved profiles. Library preparation and sequencing were carried out by Novogene (Beijing, China). Raw sequencing data (FASTQ files) were aligned to the reference genome using TopHat v2.0.12, and a splice‐junction database was generated from the corresponding gene‐model annotation file. Gene‐level read counts were obtained with HTSeq v0.6.1, and expression levels were normalized as fragments per kilobase of transcript per million mapped reads (FPKM). Differential gene‐expression and clustering analyses were performed using the ClustVis web server [73], and functional enrichment was assessed with Gene Set Enrichment Analysis (GSEA) [74]. The bulk RNA‐seq data have been deposited in the Gene Expression Omnibus (GEO) under accession number GSE217290.

### 4.7 Agent and Drug Administration

Mice were subcutaneously injected with three escalating doses (30 µg/kg, 70 µg/kg, 100 µg/kg) of resiniferatoxin (RTX; AG‐CN2‐0534, Adipogen Life Sciences) over three consecutive days. Four weeks after the initial injection, mice were exposed to cold or subjected to IF. To manipulate TRPM8 and TRPV1 signaling in the inguinal WAT, AMG333 (6874, Tocris) and AMG9810 (2316, Tocris) were injected into the inguinal WAT. To block WAT browning during cold exposure, CGRP (C0167, Sigma‐Aldrich) was injected into the inguinal WAT. To disrupt CGRP function, the CGRP receptor antagonist BIBN4096 (SML2426, Sigma‐Aldrich) was prepared by dissolving it in DMSO. Mice were administered intraperitoneal injections of 1 mg/kg BIBN4096 on Days 1 and 4 using a 10% Tween 80‐PBS solution. To disrupt Gαi function, the Gαi inhibitor 0990CL was prepared by dissolving it in DMSO and diluting it in corn oil to a final volume of 100 µL per 20 g body weight. Male *LepR‐Cre;tdTomato* mice (6–8 weeks old) were administered intraperitoneal injections of 0.010 mg/kg 0990CL on Day 0 and Day 2. On Day 3, mice were euthanized, and inguinal white adipose tissue was collected for immunofluorescence staining of pCREB and UCP1. Inguinal WAT injection was performed using the intra‐fat injection method, following a previously described protocol [75]. Briefly, mice were anesthetized, and the target area was prepared by removing the majority of hair. A dorsal incision was made on the skin to access the fat pad directly. Care was taken to ensure proper exposure of the fat pad. The drug or substance of interest was injected directly into the exposed inguinal WAT using the intra‐fat injection technique. Injections were administered unilaterally into inguinal WAT, and the contralateral depot served as the untreated control. After injection, the incision was closed with sterile wound clips and 10 mg/kg enrofloxacin was applied. Thirty minutes after local injection of CGRP, AMG333, or AMG9810, mice were exposed to cold or subjected to IF. The detailed protocols of agents or drugs used to manipulate CGRP signaling are listed in Table S1.

### 4.8 Histology and Immunostaining

Mice were terminally anesthetized with isoflurane. iWAT was collected and post‐fixed in 4% paraformaldehyde overnight at 4°C. Tissues were paraffin‐embedded, sectioned at 5 µm, mounted on glass slides, and stained with hematoxylin and eosin. Multilocular adipocytes were identified morphologically in the indicated high‐power fields.

Frozen sections were prepared for immunostaining. In brief, 16 µm sections were blocked with 10% BSA in 1% Triton X‐100/PBS for 30 min at room temperature. Sections were incubated with the following primary antibodies diluted in 2% BSA in 0.05% Triton X‐100/PBS overnight at 4°C: anti‐LepR (1:10, 20966‐1‐AP, Proteintech), anti‐COX4 (1:50, GTX114330, Genetex), anti‐UCP1 (1:250, ab10983, Abcam), anti‐CD137 (10 µg/mL, AF937, R&D), anti‐TMEM26 (1:25, NBP2‐27334, Novus Biologicals), anti‐PGC‐1α (1:50, bs‐1832, Bioss), anti‐caspase‐3 (1:400, 9661, Cell Signaling), anti‐tyrosine hydroxylase (1:100, AB152, Millipore), anti‐CGRP (1:50, bs‐0791, Bioss; 1:500, ab36001, Abcam), anti‐TRPM8 (1:200, GTX54866, Genetex), anti‐TRPV1 (1:200, GTX54762, Genetex), anti‐CRLR (1:50, MAB10044, R&D), anti‐Gαi (1:50, 5290S, Cell Signaling), anti‐AC (1:50, sc‐377243, Santa Cruz), anti‐RAMP1 (1:50, GTX108525, Genetex), anti‐RCP (1:250, ab139264, Abcam), anti‐Sca‐1 (1:50, ab109211, Abcam), anti‐EMCN (1:50, sc‐65495, Santa Cruz), anti‐norepinephrine (1:500, IS1042, Immusmol SAS), anti‐PGP9.5 (1:100, GTX634797, Genetex), anti‐F4/80 (1:50, GTX26640, Genetex), anti‐perilipin 1 (PLIN1; 1:100, GTX130139, GeneTex), anti‐SDF1 (CXCL12; 1:100, GTX116092, GeneTex), anti‐PDGFRα (1:1000, clone D1E1E, Cell Signaling Technology), anti‐PRDM16 (1:100, GTX03275, GeneTex), and anti‐pCREB (1:50, GTX53975, Genetex). Sections were washed three times with 0.05% Triton X‐100/PBS and incubated with secondary antibodies diluted in 2% BSA in 0.05% Triton X‐100/PBS for 1 h at room temperature. Secondary antibodies used were as follows: goat anti‐rabbit‐DyLight 650 (1:50, A120‐101D5, Bethyl Laboratories), goat anti‐rabbit IgG antibody‐FITC (1:50, A120‐201F, Bethyl Laboratories), anti‐rabbit‐Dylight 488 (1:100, GTX213110‐04, Genetex), goat anti‐mouse IgG antibody –DyLight 488 (1:100, GTX213111‐04, Genetex), goat anti‐rat IgG antibody‐pre‐adsorbed‐Cy5 (1:100, GTX26565, Genetex), and anti‐goat‐Alexa 488 (1:200, ab150129, Abcam). Images were acquired with a Leica SP2/SP8X confocal spectral microscope and an ANDOR Dragonfly high‐speed confocal system at the Medical Research Core Facilities, Office of Research & Development, China Medical University.

### 4.9 In Vitro Differentiation

For adipogenic induction, mouse LepR‐MSCs were cultured in growth medium at a density of 10^4^ cells/cm^2^ and then the medium was replaced with adipogenic base medium containing αMEM supplemented with 10% FBS, 100 U/mL penicillin, 100 µg/mL streptomycin, 0.25 µg/mL amphotericin B, 10^−7^ M dexamethasone (D1756, Sigma‐Aldrich) and 5 µg/mL insulin (I0516, Sigma‐Aldrich) with the addition of modulators including 0.5 mM 3‐isobutyl‐1‐methylxanthine (IBMX; I5879, Sigma‐Aldrich), 0.5 mM IBMX with 500 nM CGRP, 500 nM CGRP, 20 µM PKA inhibitor (PKI; A1117, APExBIO) or 20 µM PKI with 500 nM CGRP, and cultured for 5–7 days until the appearance of morphologic features of adipogenic differentiation. Adipogenic differentiation was detected by staining with Oil Red O (O9755, Sigma‐Aldrich) and absorbance values were measured at 510 nm after extraction with isopropanol. To measure PKA activity during adipogenesis in the presence of different modulators, the PKA kinase activity assay kit (ab139435, Abcam) was used and absorbance values were measured at 450 nm.

### 4.10 Immunoblotting

Adipose Cells Differentiated From LepR‐MSCs Were Lysed on Ice in RIPA Buffer containing protease inhibitors and phosphatase inhibitors (Bio‐PINR‐100; Bio‐PIB‐100, BioKit) With Vigorous Vortexing. Protein Lysates Were Transferred Into diluted 4X Laemmli sample buffer (1610747, Bio‐Rad) containing 2‐mercaptoethanol and heated at 100°C for 5 min. After that, lysates were separated by SDS‐PAGE and transferred to a PVDF membrane (IPVH85R, Millipore). Primary antibodies against UCP1 (GTX112784, Genetex), pCREB (06–519, Millipore), CREB (GTX112846, Genetex), and α‐tubulin (2144, Cell Signaling) were used. The secondary antibody, horseradish peroxidase‐conjugated anti‐rabbit IgG (ab6721, Abcam), was used. Blots Were Developed With the Immobilon Western Chemiluminescent HRP substrate (WBKLS0500, Millipore).

### 4.11 Growth Curve

Cell culture and seeding. Primary LepR+ MSCs were cultured in α‐MEM supplemented with 10% FBS and 1% PSA (penicillin–streptomycin–amphotericin B). Cells were seeded at 1 × 10^5^ cells per well in 6‐well plates and allowed to attach overnight (D0). From D1, cells received CGRPα at 10^−8^, 10^−7^, or 10^−6^ M, or vehicle. Treatments and medium were refreshed every 48 h throughout the assay. Cell numbers were measured daily (D1–D5) by detaching parallel wells and counting using trypan blue exclusion. For each condition and time point, n = 3 independent wells were analyzed. Data are presented as mean ± SEM.

### 4.12 Cell‐Cycle Analysis by PI Staining

LepR+ cells were isolated from *LepR‐Cre;tdTomato* mice and referred to as LepR‐MSCs. Adipogenic differentiation was induced with 10^−7^ M dexamethasone and 5 µg mL^−1^ insulin (Adipose base) in the presence of 0.5 mM IBMX, with or without CGRP (10^−8^–10^−6^ M) as indicated for 5 days. Cells were washed in PBS and fixed with methanol for 10 min at room temperature. Fixed cells were permeabilized with 0.1% Triton X‐100 in PBS for 5 min, washed, and stained with propidium iodide (PI, 48 µg mL^−1^) containing DNase‐free RNase A (48 µg mL^−1^) for 20 min in the dark at room temperature. Data were acquired on a BD Accuri C6 Plus (BD Biosciences). PI fluorescence was collected on FL2 (585/40 nm) and recorded on a linear scale using BD Accuri C6 Plus software (CFlow Plus). Analyses were performed in FlowJo v10.8.2 and GraphPad Prism v5. Debris was excluded by FSC‐A vs SSC‐A; singlets were gated using FSC‐A vs FSC‐H (doublet discrimination).

### 4.13 Live/Dead Cell Viability Assay

Cell viability was assessed using the LIVE/DEAD Cell Imaging Kit (488/570; Invitrogen, R37601) according to the manufacturer's instructions. Cells were stained with the live/dead dye mixture and imaged by fluorescence microscopy. Viable and dead cells were identified by green and red fluorescence signals, respectively.

### 4.14 *Lepr* siRNA Electroporation

Primary mouse bone marrow‐derived MSCs were transfected with scrambled control siRNA or *Lepr*‐targeting siRNA using the Neon NxT Electroporation System and a 10‐µL Neon NxT Kit (Thermo Fisher Scientific). Cells at 70%–90% confluence were detached, washed with Ca^2^^+^/Mg^2^^+^‐free PBS, and resuspended in Resuspension R Buffer at a density of 1–5 × 10^7^ cells/mL. For each reaction, 2 × 10^5^ cells were mixed with 0.5 µL of 20 µM siRNA in a final volume of 10 µL, corresponding to a final siRNA concentration of 1 µM. The *Lepr*‐targeting siRNA comprised sense 5′‐GGGCUUCUGAAGCAAUUGUTT‐3′ and antisense 5′‐ACAAUUGCUUCAGAAGCCCTT‐3′ sequences. Electroporation was performed at 1,400 V using two 20‐ms pulses. Immediately after electroporation, cells were transferred to prewarmed growth medium without antibiotics and allowed to recover for 24 h at 37°C before subsequent experiments. *Lepr* knockdown was confirmed by RT‐qPCR and immunoblotting (Figure S13).

### 4.15 Whole‐Mount Staining

Whole‐mount tissue was prepared using a clearing‐free protocol with Z‐depth reduction for processing intact inguinal WAT [76]. Briefly, mice were sacrificed, and inguinal WAT was carefully excised. Inguinal WAT was fixed in 2% paraformaldehyde at 4°C for 8–24 h depending on tissue thickness. After fixation, Z‐depth reduction was performed by clamping inguinal WAT between two large glass slides for 90 min at 4°C. Z‐depth‐reduced inguinal WAT was treated in 2.5% BSA with 1% Triton X‐100 for 16–24 h at 4°C for blocking and permeabilization. Then, autofluorescence was quenched as follows. Tissues were dehydrated stepwise in increasing concentrations of MeOH diluted in 1X PBS (50% MeOH for 1 h; 80% MeOH for 1 h; 100% MeOH for 2 h, replaced with fresh solution after 1 h) at ∼20°C. Tissues were bleached in 5% H_2_O_2_ in 20% DMSO/MeOH for 16–24 h at 4°C. This was followed by rehydration in decreasing concentrations of MeOH diluted in 1X PBS (100% MeOH for 2 h, replaced with fresh solution after 1 h; 20% DMSO/MeOH for 1 h; 80% MeOH for 1 h; 50% MeOH for 1 h; 1X PBS for 2 h, replaced with fresh solution after 1 h) at ∼20°C. Tissues were incubated with primary antibody for 48 h at 4°C, followed by washing and incubation with secondary fluorescent antibodies overnight. Lipid droplets were labeled by Nile Red (N3013, Sigma‐Aldrich). Then, tissues were incubated with 100 ng/mL DAPI for 1 h at room temperature. Afterwards, tissues were placed on large glass slides with a few drops of glycerol‐based mounting medium added and coverslips were placed on top, weighted for 2 days, and then sealed. Images were acquired using an ANDOR Dragonfly high‐speed confocal system. Confocal images of inguinal WAT were then analyzed for the spatial association between CGRP+ sensory fibers and TH+ sympathetic fibers. ROIs were manually defined around TH+ neurovascular bundle regions based on the local TH^+^ nerve axis and shared neurovascular trajectory. Within each ROI, CGRP^+^ fibers were assessed for alignment and close spatial association with TH^+^ neurovascular axes, rather than true pixel‐level colocalization. For each animal, three high‐power fields from a single whole‐mount tissue section were analyzed (n = 3 animals).

### 4.16 Super‐Resolution Confocal Imaging

Super‐resolution confocal images were acquired using a Nikon Ti2 Eclipse microscope equipped with an AXR confocal system and a Nikon Spatial Array Confocal (NSPARC) detector. Images were acquired with a 20 × objective under NIS‐Elements software. DAPI, CGRP and TH signals were excited using 405, 488, and 647 nm laser lines, respectively. Images were processed using NIS‐Elements for representative visualization of TH+ sympathetic and CGRP+ sensory fibers in neurovascular and parenchymal regions of inguinal WAT.

### 4.17 Nerve‐Fiber Density Quantification

In each three‐dimensional image, three small cuboidal volumes were selected per sample. The Filament tool [77] was used to reconstruct TH‐positive sympathetic and CGRP‐positive sensory fibers and calculate total fiber length within each volume. Fiber density was expressed as total traced fiber length (µm) divided by regional volume (µm^3^). Three high‐power fields were averaged for each animal, and each animal was treated as one biological replicate.

### 4.18 Intraganglionic Dorsal Root Ganglia (DRG) Injection

The AAV expression vector contained a human U6 promoter to drive expression of the shRNA targeting CGRP and a PGK promoter to drive the GFP reporter. Serotype 9 AAV(AAV9) vectors encoding mouse CGRP shRNAs (AAV‐shCGRP

TL500251A‐AGGCAGTGCCTTTGAGGTCAATCTTGGAA) and AAV‐Scramble (TR30021 – GCACTACCAGAGCTAACTCAGATAGTACT) were produced via the AAV Core of IBMS, Academia Sinica. The intraganglionic DRG injection was conducted following a previously reported method [14]. A midline incision was made along the dorsal skin to access the underlying dorsal muscles, which were carefully separated along the vertebral column to expose the DRGs. The DRGs were carefully isolated at the T13 and L1 vertebral levels. 200 nL of AAV9 viral vector (at a concentration of 1 × 10^13^ viral genomes/mL) in PBS containing 0.3% Fast Green FCF (Sigma‐Aldrich) was injected into each ganglion through a pulled glass pipette with a Nanoliter Injector (RWD). Following the injection, the dorsal muscles and skin were sutured, and the animal was permitted to recover from anesthesia.

### 4.19 Quantitative Reverse‐Transcription PCR (RT‐qPCR)

Mice exposed to cold, subjected to IF, or maintained under control conditions (Ctrl) were euthanized, and inguinal WAT was collected. Tissues were lysed and homogenized in TRIzol reagent (15596018, Ambion) and were phase‐separated by bromochloropropane (BCP; BP 151, Molecular Research Center). RNA was precipitated from the aqueous phase using isopropanol and washed with ethanol. After removing the ethanol followed by centrifugation, RNA was briefly air‐dried and solubilized in DEPC water. Total RNA was subjected to reverse transcription (MMLV Reverse Transcription Kit; PT RT KIT, Protech Technology) and then RT‐qPCR using SYBR Green (iQ SYBR Green Supermix; 1708882, Bio‐Rad) on a CFX96 Optical Reaction Module (Bio‐Rad). Relative transcript abundance was normalized to *Gapdh* and calculated using the 2^‐ΔΔCt method. Primer sequences are listed in Table S2.

### 4.20 Proximity Ligation Assay (PLA)

PLA was performed according to the manufacturer's Duolink PLA fluorescence protocol (DUO92004, Sigma‐Aldrich). In brief, the frozen sections were permeabilized by 1% Triton X‐100/PBS for 30 min at room temperature. Sections were incubated with blocking buffer for 60 min at 37°C. The following primary antibodies were diluted in Duolink antibody diluent and incubated with the sections overnight at 4°C: anti‐CRLR (1:50, MAB10044, R&D), anti‐CGRP (1:50, bs‐0791, Bioss), anti‐Gαi (1:50, 5290, Cell Signaling), and anti‐AC (1:50, sc‐377243, Santa Cruz). First, slides were incubated with Duolink PLA probes for 60 min at 37°C. Then, slides were incubated with Duolink PLA ligation buffer for 30 min at 37°C. Last, slides were incubated with Duolink PLA amplification buffer for 100 min at 37°C. Slides were mounted using Duolink PLA mounting medium with DAPI. Images were acquired with a Leica SP2/SP8X confocal spectral microscope at the Medical Research Core Facilities, Office of Research & Development, China Medical University.

### 4.21 In Vivo Macrophage Depletion Protocol

In the experimental procedure, we administered liposome‐encapsulated clodronate (referred to as Clodronate) or liposomes as a control (referred to as the vehicle, CLD‐8901 from Encapsula NanoSciences) to mice via intravenous (i.v.) injection. The injection volume for both substances was 0.2 mL, which corresponded to a dosage of 1 mg per mouse. The liposome‐encapsulated clodronate and the liposomes used as a vehicle control were administered 2 days before the initiation of the IF protocol.

### 4.22 Analyses of Publicly Available Single‐Cell RNA‐Sequencing Data

Single‐cell RNA‐sequencing (scRNA‐seq) data from GSE176067 were analyzed using the R package Seurat [78] (version 5), and the authors provided metadata on cell type annotations. To identify the differentially expressed genes in each of the subclusters, the FindAllMarkers function was applied with a minimum threshold of 30% of cells in the cluster expressing the gene and a minimum log2FC threshold of 1. Single‐nucleus RNA sequencing data from human BAT were obtained from E‐MTAB‐8564 [52]. The datasets can be explored interactively at https://batnetwork.org/.

### 4.23 Statistical Analysis

Data are presented as mean ± SEM. For animal experiments, each plotted data point represents one independent mouse. When multiple imaging fields were acquired from one mouse, field‐level measurements were averaged to generate one value for that mouse and were not treated as independent replicates. Paired two‐tailed Student's t tests were used for within‐animal comparisons of injected and contralateral inguinal WAT depots; other two‐group comparisons used two‐tailed unpaired Student's t tests. Comparisons among multiple groups used one‐way ANOVA with Tukey's multiple‐comparison test. Growth curves were analyzed by two‐way ANOVA with treatment and time as factors, followed by Tukey's multiple‐comparison test. Statistical analyses were performed using GraphPad Prism 5, and p < 0.05 was considered significant. Exact sample sizes and panel‐specific tests are reported in the figure legends.

## Author Contributions

Y.T.Y. validated and extended the data, performed experiments, analyzed the data and wrote the paper. M.C. validated and extended the data, designed and performed experiments, analyzed the data and wrote the paper. Y.T.K. and D.C.W. performed experiments, analyzed the data and wrote the methods. H.T.C. and S.L.L. contributed to the genetic ablation and lineage tracing experiments in mice. M.C.H. contributed to concept development and wrote the paper. S.C.H. wrote the proposal, designed and supervised the study, analyzed the data and wrote the paper.

## Funding

Grants supported by Minister of Science and Technology (NSCT 109‐2314‐B‐039 ‐009 ‐MY3; MOST 111‐2326‐B‐039‐001; NSTC 112‐2320‐B‐039 ‐007 ‐MY2; NSTC 113‐2314‐B‐039‐065) and Integrative Stem Cell Center, China Medical University (CMU110‐Z‐05; CMU111‐N‐29) and China Medical University Hospital (DMR‐111‐233). The funding sources had no involvement in study design; data collection, analysis, or interpretation; writing of the report; or the decision to submit the article for publication.

## Conflicts of Interest

The authors declare no conflicts of interest.

## Supporting information




**Supporting File**: advs78714‐sup‐0001‐SuppMat.docx.

## Data Availability

The data that support the findings of this study are openly available in NCBI Gene Expression Omnibus (GEO) at https://www.ncbi.nlm.nih.gov/geo/, reference number GSE217290; GSE216925.
