# Supplementary material for: LepR‐Lineage Cells Represent the Main Target of the Nociceptive CGRP Pathway in White Adipose Tissue Browning
Source: Adv Sci (Weinh). 2026 Nov 9:e78714. Online ahead of print. doi: 10.1002/advs.78714 (PMC13748883; doi:10.1002/advs.78714)
Supplement: Supplementary file 1 [file ADVS-9999-e78714-s001.docx]

**Supporting Information**

**LepR-lineage cells represent the main target of the nociceptive CGRP pathway in white adipose tissue browning**

Yu-Ting Yen, May Chien, Yan-Tung Kiu, Ru-Sing Lee, Hui-Teng Cheng, Shuei-Liong Lin, Dong-Chuan Wu, I-Hsien Teng, Yi-Wen Lin, Yi-Hung Chen, Mien-Chie Hung, Shih-Chieh Hung*

Supplementary Materials for this manuscript include the following:

Figures S1 to S14

Tables S1 to S2

**Figure S1 Responses of LepR-lineage cells to cold exposure and intermittent fasting.**

(a) Representative confocal immunofluorescence images of tdTomato with CD137, TMEM26, COX4, or PGC-1α in iWAT from *LepR-Cre;*Rosa26-LSL-tdTomato mice under Control (Ctrl), Cold, and intermittent fasting (IF) conditions. Arrows indicate double-positive cells. The percentage of tdTomato-positive cells expressing each marker is shown; n.d. indicates not detected. (b) Early IF response at days 1 and 3. Representative tdTomato and cleaved caspase-3 immunofluorescence and the percentage of cleaved caspase-3-positive cells among tdTomato-positive cells are shown. (c) Experimental timeline and representative images after 1 week of IF and after 2 or 4 weeks of refeeding. tdTomato-positive cells were nearly undetectable after 1 week of IF and reappeared after refeeding; cleaved caspase-3 signals were minimal during recovery. Each plotted point represents one mouse. Data are mean ± SEM. One-way ANOVA with Tukey's multiple-comparison test or two-tailed unpaired Student's t test was used as indicated. **P* < 0.05, ***P* < 0.01, ****P* < 0.001. Scale bars, 50 μm.

**Figure S2 Whole-iWAT bulk RNA-seq shows treatment-associated remodeling of adrenergic and cAMP-PKA signaling.**

(a) GSEA of the PANTHER pathway MM_BETA2_ADRENERGIC_RECEPTOR_SIGNALING using the ranked Cold-versus-Control differential-expression list from whole-iWAT bulk RNA-seq. (b) Heatmap of the displayed pathway components across Control, Cold, and IF biological replicates; rows show z-scored log2 expression.

**Figure S3 Sympathetic and CGRP-containing sensory fibers in iWAT.**

(a) TH, CGRP, and CD31 immunofluorescence in neurovascular-bundle and parenchymal regions. (b) TH and CGRP immunofluorescence with boxed regions shown at higher magnification. (c) Super-resolution images resolving closely aligned but distinct TH-positive sympathetic and CGRP-positive sensory fibers. (d) CD137, TMEM26, and COX4 immunofluorescence under Ctrl, Cold, Cold/RTX, IF, and IF/RTX conditions, with quantification. Each plotted point represents one mouse. Data are mean ± SEM. One-way ANOVA with Tukey's multiple-comparison test was used. **P* < 0.05, ****P* < 0.001. Scale bars, 50 μm (a,b,d) and 20 μm (c).

**Figure S4 Body weight and rectal temperature during nociceptive and CGRP-pathway perturbations.**

Body weight and rectal temperature during (a) Ctrl, Ctrl/RTX, Cold, Cold/RTX, IF, and IF/RTX; (b) Cold, Cold/CGRP, and Cold/AMG333; (c) Ctrl, Ctrl/BIBN4096, IF, and IF/BIBN4096; (d) IF and IF/AMG9810; (e) IF/vehicle and IF/clodronate; (f) Ctrl, Ctrl/CGRPα, Cold, Cold/CGRPα, IF, and IF/CGRPα; and (g) Ctrl/AAV-Scramble, Ctrl/AAV-shCGRPα, Cold/AAV-Scramble, Cold/AAV-shCGRPα, IF/AAV-Scramble, and IF/AAV-shCGRPα groups. Each plotted point represents one mouse. Data are mean ± SEM. One-way ANOVA with Tukey's multiple-comparison test or two-tailed unpaired Student's t test was used as indicated. **P* < 0.05, ***P* < 0.01, ****P* < 0.001.

**Figure S5 CGRP-associated signaling and WAT browning in LepR-lineage cells.**

Representative anti-tdTomato immunohistochemistry and tdTomato/UCP1 confocal immunofluorescence in iWAT from *LepR-Cre;*Rosa26-LSL-tdTomato mice under (a) Ctrl, Cold, Cold/RTX, IF, and IF/RTX; (b) Cold, Cold/CGRP, and Cold/AMG333; (c) Ctrl, Ctrl/BIBN4096, IF, and IF/BIBN4096; and (d) IF and IF/AMG9810 conditions. Quantification beneath each panel uses the endpoint identified on the corresponding plot. Within-lineage percentages were calculated only when a tdTomato-positive denominator was detectable. Each plotted point represents one mouse. Data are mean ± SEM. One-way ANOVA with Tukey's multiple-comparison test or two-tailed unpaired Student's t test was used as indicated. **P* < 0.05, ***P* < 0.01, ****P* < 0.001. Scale bars, 50 μm.

**Figure S6 CGRP attenuates PKA and CREB signaling in bone marrow-derived LepR+ MSCs.**

Primary mouse bone marrow-derived LepR+ MSCs were differentiated in adipogenic base medium with the indicated combinations of IBMX, CGRP, and PKA inhibitor (PKI). (a) Oil Red O staining. (b) Oil Red O absorbance at 510 nm. (c) PKA activity measured at 450 nm. (d) Immunoblotting and densitometry for pCREB, total CREB, UCP1, and α-tubulin. CGRP reduced IBMX-induced PKA activity and CREB phosphorylation; the decrease in UCP1 between IBMX and IBMX plus CGRP did not reach statistical significance. (e) Cell numbers during 5 days of CGRP treatment. (f) DNA-content analysis showing the sub-G1 fraction after 5 days. (g) Viability staining of remaining cells following CGRP treatment. Representative fluorescence images of MSC stained with Calcein-AM (live cells, green) and ethidium homodimer-1 (EthD-1; dead cells, red). Each plotted point represents one independent culture. Data are mean ± SEM. Panels b-d and f were analyzed by one-way ANOVA with Tukey's test; panel e was analyzed by two-way ANOVA with treatment and time as factors followed by Tukey's test. **P* < 0.05, ***P* < 0.01, ****P* < 0.001. Scale bar, 50 μm.

**Figure S7 TRP-family transcript profiles in whole iWAT.**

RT-qPCR analysis of Trpc, Trpm, *Trpa1*, and Trpv transcripts in whole iWAT from Ctrl, Cold, and IF mice. Relative expression was normalized to *Gapdh* and calculated using the 2^-ΔΔCt method. n = 3-4 mice per group. Data are mean ± SEM. Because whole-iWAT RNA includes neuronal and non-neuronal cell types, these profiles do not identify the cellular source of the transcripts.

**Figure S8 CGRP-receptor components and vascular localization of LepR-lineage cells.**

Representative iWAT immunofluorescence in *LepR-Cre;*Rosa26-LSL-tdTomato mice for (a) tdTomato, CRLR, and RAMP1 and (b) tdTomato, CRLR, and RCP. Arrows indicate receptor-component signals associated with tdTomato-positive cells. The percentages of tdTomato-positive and tdTomato-negative cells positive for the indicated combinations are quantified. (c) tdTomato, EMCN, and Sca-1 immunofluorescence with higher-magnification views and arrows highlighting the close spatial association of tdTomato-positive cells with EMCN-positive sinusoidal vessels. This panel defines vascular localization rather than lineage identity. Each plotted point represents one mouse. Data are mean ± SEM. One-way ANOVA with Tukey's multiple-comparison test was used. ***P* < 0.01, ****P* < 0.001. Scale bars are shown in the images.

**Figure S9 Molecular proximity among CGRP-receptor and Gαi pathway components.**

(a) Gαi and adenylyl cyclase (AC) immunofluorescence under the indicated conditions. (b) PLA analysis of CRLR/CGRP, CRLR/Gαi, and AC/Gαi proximity. PLA signals indicate molecular proximity and do not by themselves establish direct binding or pathway activation. (c) Gαi, Gαs, and tdTomato immunofluorescence. Arrows indicate positive signals. Each plotted point represents one mouse. Data are mean ± SEM. Two-tailed unpaired Student's t tests were used for the indicated pairwise comparisons. **P* < 0.05, ***P* < 0.01, ****P* < 0.001. Scale bars are shown in the images.

**Figure S10 Gαi inhibition affects pCREB and UCP1 under Control, Cold, and IF conditions.**

Representative pCREB, UCP1, and DAPI immunofluorescence in Ctrl, Ctrl/0990CL, Cold, Cold/0990CL, IF, and IF/0990CL groups. Quantification of the endpoints indicated on the plots is shown. Gαi inhibition increased pCREB and UCP1 under Ctrl and IF conditions but produced no significant additional increase during Cold, when both markers were already elevated. Intracellular cAMP and pathway saturation were not measured. Each plotted point represents one mouse. Data are mean ± SEM. Two-tailed unpaired Student's t tests were used for the indicated pairwise comparisons. **P* < 0.05, ***P* < 0.01, ****P* < 0.001. Scale bars, 50 μm.

**Figure S11 Expression of *LEPR* and CGRP-receptor-component genes in human adipose datasets.**

(a,b) UMAP representations of published human subcutaneous adipose tissue (SAT; n = 86,064 cells) and visceral adipose tissue (VAT; n = 80,085 cells), with expression of *LEPR, CALCRL, CRCP*, *RAMP1*, *RAMP2*, and *RAMP3*. GEO accession: GSE176067. (c) Dot plot of these genes across annotated endothelial clusters. Dot size indicates the percentage of cells expressing each gene, and color indicates normalized expression. (d) UMAP representation of 36,590 nuclei from human brown adipose tissue with the same gene overlays. Accession: E-MTAB-8564. These panels show marker correspondence and do not establish functional equivalence to mouse LepR-lineage stromal/progenitor cells.

**Figure S12** **Single-transgenic controls show no detectable cell-associated tdTomato signal in iWAT.**

Representative fluorescence images of iWAT from LepR-Cre-only mice, tdTomato-reporter-only mice, and *LepR*-Cre;Rosa26-LSL-tdTomato mice. No detectable cell-associated tdTomato signal was observed in the single-transgenic controls. DAPI labels nuclei; boxed regions indicate magnified areas in the double-transgenic positive example. Scale bars are shown in the images.

**Figure S13 Validation of *Lepr* knockdown in bone marrow-derived MSCs following siRNA transfection.**

(a) Relative *Lepr* mRNA expression in primary mouse bone marrow-derived MSCs transfected with scrambled control siRNA or *Lepr*-targeting siRNA. The *Lepr*-targeting duplex comprised sense 5′-GGGCUUCUGAAGCAAUUGUTT-3′ and antisense 5′-ACAAUUGCUUCAGAAGCCCTT-3′ sequences. Expression was normalized to *Gapdh* and calculated using the 2^-ΔΔCt method. (b) Representative immunoblots and densitometric quantification of LepR protein in bone marrow-derived MSCs following the indicated siRNA transfection. Arrows indicate the expected LepR band at approximately 100-150 kDa; β-actin served as the loading control. Each plotted point represents one biological replicate from an individual mouse. Data are mean ± SEM. Two-tailed unpaired Student's t tests were used. **P* < 0.05, ****P* < 0.001.

**Supplemental Figure S14. Validation of LepR-lineage cell depletion following diphtheria toxin treatment.** Representative fluorescence images showing tdTomato-labeled LepR-lineage cells (red) and DAPI-stained nuclei (blue) in iWAT from DT-treated LepR-Cre;Rosa26-tdTomato mice, vehicle-treated LepR-Cre;Rosa26-tdTomato/DTR mice, and DT-treated *LepR*-Cre;Rosa26-tdTomato/DTR mice. Each dot represents one mouse (n = 2 per group). Data are presented as mean ± SEM. Statistical significance was assessed by one-way ANOVA followed by Tukey’s multiple-comparisons test. *P < 0.05.

**Table S1 Agonist and antagonist administration method, dosage and frequency.**

| **Item** |  | **Drug** | **Injection method** | **Dose (concentration)** | **Frequency/time point** | **Treatment protocol** | **UCP1** |
| --- | --- | --- | --- | --- | --- | --- | --- |
| CGRP receptor | agonist | CGRPα (C0167, Sigma-Aldrich) | Direct injection into inguinal WAT at 12 sites, 1 μL per site | 0.2 mg/kg (0.5 μg /μL) | Once, 30 min before the start of the treatment protocol | Cold/IF | ↓ |
|  | antagonist | BIBN4096 (SML2426, Sigma-Aldrich) | Intraperitoneal injection, 100 μL per injection | 1 mg/kg (0.03 mg/100 μL) | Twice, at Day 1 and Day 4 during the treatment protocol | Ctrl/IF | ↑ |
| TRPV1 | agonist | Resiniferatoxin (RTX, AG-CN2-0534, AdipoGen Life Sciences) | Subcutaneous injections on three consecutive days | Day1 (30 μg/kg), Day2 (70 μg/kg), Day3 (100 μg/kg), (1μg/μL) | Three injections, 4 weeks before the start of the treatment protocol | Cold/IF | ↓ |
|  | antagonist | AMG9810 (2316, Tocris) | Direct injection into inguinal WAT at 12 sites, 3.75 μL per site | 30 mg/kg (20 μg /μL) | Once, 30 min before the start of the treatment protocol | Cold/IF | ↑ |
| TRPM8 | antagonist | AMG333 (6874, Tocris) | Direct injection into inguinal WAT at 15 sites, 1 μL per site | 1 mg/kg (2 μg/μL) | Once, 30 min before the start of the treatment protocol | Cold | ↓ |
| IF: intermittent fasting | | | | | | | |

**Table S2 Primer sequences for RT-qPCR.**

| **Gene** | **Forward primer (5’ – 3’)** | **Reverse primer (5’ – 3’)** |
| --- | --- | --- |
| *Gapdh* | CAAGCTCATTTCCTGGTATG | TCTTGCTCAGTGTCCTTGCT |
| *Lepr* | AGGGCCTGAATTTTGGAGAAA | ACGATGCTTCACCACGTACCT |
| *Ucp1* | GGCCTCTACGACTCAGTCCA | TAAGCCGGCTGAGATCTTGT |
| *Trpc1* | CAAGATTTTGGGAAATTTCTGG | TTTATCCTCATGATTTGCTAT |
| *Trpc2* | GATCCGGTTCATGTTCATCCT | GAGCGAGCAAACTTCCACTC |
| *Trpc3* | TGACTTCCGTTGTGCTCAAATATG | CCTTCTGAAGCCTTCTCCTTCTGC |
| *Trpc4* | TCTGCAGATATCTCTGGGAAGGATGC | AAGCTTTGTTCGAGCAAATTTCCATTC |
| *Trpc5* | ATCTACTGCCTAGTACTACTGGCT | CAGCATGATCGGCAATGAGCTG |
| *Trpc6* | AAAGATATCTTCAAATTCATGGTC | CACGTCCGCATCATCCTCAATTTC |
| *Trpc7* | CGTGCTGTATGGGGTTTATAATG | GCTTTGGAATGCTGTTAGAC |
| *Trpv1* | GCATCTTCTACTTCAACTTCTTCGTC | CCACATACTCCTTGCGATGGC |
| *Trpv2* | CGGCACTTCCTCTCT | GTCGGTCACGGTCAA |
| *Trpv3* | CAAGGACTGCCACCACCATC | CATCACAGTTGCCAGAGAGG |
| *Trpv4* | GAGTCCTCAGTAGTGCCTGG | CAACAAGAAGGAGAGCAGTC |
| *Trpv5* | CGTTGGTTCTTACGGGTTGAA | GTTTGGAGAACCACAGAGCCTCTA |
| *Trpv6* | AACCAGCCTTCCACC | CCTCCATTAGCACCA |
| *Trpm1* | TATCCTATGACACCAAGCCA | GGTCTTCCTTATTCTCCACG |
| *Trpm2* | CAGATCCCAACCTACATTGACG | GAAGGTGTAGTTGAACATGGCGA |
| *Trpm3* | CCTGTTCTTCTGGCA | GCTTCTCTGGCTCCT |
| *Trpm4* | CCCTGAGGATGGTGTTGAGT | AGGAGCACTGGGATGTCAAT |
| *Trpm5* | CAGATACTGAGGATGGCTGG | GGATCTTGGTGGATGTGCTA |
| *Trpm6* | CCAGGTGCCGGTAATAACA | CTCTTGTGGCTGCCTTAGGT |
| *Trpm7* | CGGAGCTGGTCGCACAATTA | CCTGGAAGACATCTGTGAGG |
| *Trpm8* | TCCACGAGACTTCTTCACTT | GTGTATCTGAGGTCAGTCTT |
| *Trpa1* | TGCTGCAGAAAAAAATCAAGTTGA | CCTTGGCTGAGAAGAAACTTTACACT |
